# Addressing the most neglected diseases through an open research model: The discovery of fenarimols as novel drug candidates for eumycetoma

**DOI:** 10.1371/journal.pntd.0006437

**Published:** 2018-04-26

**Authors:** Wilson Lim, Youri Melse, Mickey Konings, Hung Phat Duong, Kimberly Eadie, Benoît Laleu, Benjamin Perry, Matthew H. Todd, Jean-Robert Ioset, Wendy W. J. van de Sande

**Affiliations:** 1 Erasmus MC, Department of Medical Microbiology and Infectious Diseases, Rotterdam, The Netherlands; 2 School of Chemistry, The University of Sydney, Sydney, Australia; 3 Medicines for Malaria Venture (MMV), Geneva, Switzerland; 4 Drugs for Neglected Diseases *initiative* (DND*i*), Geneva, Switzerland; University of Tennessee, UNITED STATES

## Abstract

Eumycetoma is a chronic infectious disease characterized by a large subcutaneous mass, often caused by the fungus *Madurella mycetomatis*. A combination of surgery and prolonged medication is needed to treat this infection with a success rate of only 30%. There is, therefore, an urgent need to find more effective drugs for the treatment of this disease. In this study, we screened 800 diverse drug-like molecules and identified 215 molecules that were active *in vitro*. Minimal inhibitory concentrations were determined for the 13 most active compounds. One of the most potent compounds, a fenarimol analogue for which a large analogue library is available, led to the screening of an additional 35 compounds for their *in vitro* activity against *M*. *mycetomatis* hyphae, rendering four further hit compounds. To assess the *in vivo* potency of these hit compounds, a *Galleria mellonella* larvae model infected with *M*. *mycetomatis* was used. Several of the compounds identified *in vitro* demonstrated promising efficacy *in vivo* in terms of prolonged larval survival and/or reduced fungal burden. The results presented in this paper are the starting point of an *Open Source Mycetoma (MycetOS)* approach in which members of the global scientific community are invited to participate and contribute as equal partners. We hope that this initiative, coupled with the promising new hits we have reported, will lead to progress in drug discovery for this most neglected of neglected tropical diseases.

## Author summary

Mycetoma is a poverty-associated disease that was recently recognised as a neglected tropical disease by the World Health Organisation (WHO). This disease can be caused by either bacteria (actinomycetoma) or fungi (eumycetoma). The most common causative agent of mycetoma is the fungus *Madurella mycetomatis*. Actinomycetoma can be easily treated, but for eumycetoma, the current and only antifungal drug used is only able to successfully treat 30% of patients. Treatment often involves prolonged medication use and amputation of the affected area. This disease is disfiguring and is a social stigma for patients in endemic countries. To improve treatment for patients, we have looked at over 800 diverse drug-like molecules and compounds in hope to develop new drugs in this study. We have identified 215 compounds with activity against *M*. *mycetomatis* in vitro and several in vivo with our *Galleria mellonella* larvae model. We have chosen an open source approach with this study and placed our findings in an online database and made it available to the public. We invite the global scientific community to participate in our study and contribute as equal partners as long as an open source approach is held in hopes to fast track and boost drug discovery for Eumycetoma.

## Introduction

Mycetoma is a chronic infectious and inflammatory disease, characterised by a large subcutaneous mass and the excretion of grains, usually affecting the lower limbs [1]. This infection can be caused by either bacteria (actinomycetoma) or fungi (eumycetoma) [1] and the most common causative agent of mycetoma is the fungus *Madurella mycetomatis* [2]. Given its diverse etiology, it is not surprising that the course of treatment depends on the causative agent [3]. In general, actinomycetoma can be treated with medication only, with success rates of up to 90% [4], while for eumycetoma a combination of surgery and prolonged medication is needed [3]. Ketoconazole has been the mainstay of medical treatment for decades, given in a dose of 400–800 mg/day for a year with 50–70% success rates when combined with surgery [5, 6]. Recently, its use was restricted by the European Medicines Agency (EMA), the United States Food and Drug Administration (FDA) and the government of Sudan due to potentially fatal liver injury, drug interactions and adrenal gland problems [7, 8]. This restriction causes a dilemma, as few other options are available. Itraconazole is the most widely used alternative and in a recent study no serious hepatoxicity was noted (400 mg/day for 3 months, then 200 mg/day for 9 months) [9]. In all patients, the lesions were reduced enabling less mutilating surgery but, as is the case for ketoconazole, the fungus was still viable when isolated from surgical material [9, 10]. Other azoles have been used to some extent and with some success, but these are usually too expensive for use in endemic regions. Indeed, the only affordable drug for endemic areas was ketoconazole, at a cost of $30/month. With an average monthly income of only $60/month, itraconazole at $330/month is already considered to be too expensive for the patient and the affordability problem becomes more acute with the newer generation of antifungal agents such as voriconazole and posaconazole [11].

There is an urgent need to find an effective, safe and affordable oral antifungal agent with a short treatment duration for eumycetoma. Traditionally, the pharmaceutical industry has taken the lead in the development of novel antimicrobial drugs, but antimicrobial drug development programs have been drastically reduced in recent decades due to increased costs, decreased return on investment and the prospect of a long and expensive development process. Furthermore, drugs have never been specifically developed for neglected infectious tropical diseases such as mycetoma, due to the lack of sufficient potential return on investment. Therefore, in order to find a new, safe and effective treatment for eumycetoma, alternative approaches are necessary. A strategy that has gained much attention recently is to screen drugs already approved for other indications or drug candidates with a historical track record of development. This approach, also known as drug repurposing, is appealing as it is expected to reduce the overall costs and the timeframe required for drug development as well as de-risking the development process by capitalizing on preclinical and possibly phase I clinical data packages already available from a pre-existing drug development program [12].

The Pathogen Box is a set of 400 diverse, cherry-picked drug-like molecules previously shown to be active against pathogens causing tropical and neglected diseases. The Box includes a collection of approved drugs—the so-called reference set—used for the treatment of such diseases [13] and is made available free of charge by the Medicines for Malaria Venture (MMV) as an Open Access initiative tool to stimulate research and development on neglected diseases [12, 13]. In return, researchers are asked to share any data generated in the public domain within 2 years, creating an open and collaborative forum for infectious disease drug research. The chemical structures of the molecules in the Pathogen Box are publicly available and all compounds are annotated with valuable biological data sets arising from phenotypic screens, including cytotoxicity. Additionally, key *in vitro* and *in vivo* data related to drug metabolism and pharmacokinetics (DMPK) have recently been associated with all 400 compounds. A related MMV drug repurposing subset, the Stasis Box, has also been made available and consists of 400 compounds selected by medicinal chemistry experts out of 8000 compounds which have entered preclinical or clinical development but have been discontinued for various reasons, for example a lack of efficacy [14]. All compounds included in the Stasis Box can be purchased from commercial suppliers and have their chemical structures available in the public domain.

Here we describe the screening of the 800 drug-like compounds contained in the Pathogen and Stasis Boxes, as well as selected compounds from the fenarimol-based chemical library, for their ability to inhibit the *in vitro* growth of *M*. *mycetomatis*. The hits with greatest potential were verified through chemical resynthesis and further evaluated in our grain model in *G*. *mellonella* larvae to determine the *in vivo* efficacy against *M*. *mycetomatis* grains. Finally, we propose a strategy for executing hit-to-lead campaigns based on these results that takes a highly collaborative, community-centered approach.

## Material and methods

### Chemical libraries

The Pathogen and Stasis Boxes were kindly provided by Medicines for Malaria Venture (MMV, Geneva, Switzerland). The compounds were obtained in a 10 mM solution in dimethyl sulfoxide (DMSO) (S1 Table). The fenarimol analogues were kindly provided by DND*i via* Epichem (Perth, Australia) and dissolved in DMSO, to reach a concentration of 10 mM. The resynthesis of hit compounds, and the chemical synthesis of the novel analogues reported, are described in S2 Text.

### Mycetoma strains

In this study, *M*. *mycetomatis* genome strain MM55 [15] was used to screen the Pathogen and Stasis Boxes and to determine the IC50 and IC90 of each of the compounds with inhibitory activity at a concentration of 100 μM. *In vivo* efficacy was also determined for this strain. To assess the activity of the selected compounds against other *M*. *mycetomatis* isolates, isolates P1, MM25, MM36, MM50, MM55, MM68 and MM83 were selected [16]. To assess the activity of the fenarimol analogues, strains MM13, MM14, MM26, MM30, MM41, MM45, MM49, MM50, MM54 and MM55 were selected. Strains MM26 and MM45 belonged to Amplified Fragment Length Polymorphism (AFLP) cluster I, strains P1, MM13, MM14, MM25, MM30, MM36, MM41, MM49, MM50, MM54, MM55 and MM68 belonged to AFLP cluster II and strain MM83 belonged to AFLP cluster III [16]. All MM-isolates have been isolated from patients diagnosed with mycetoma seen in the Mycetoma Research Centre, Khartoum, Sudan; the P1 strain originated from a patient in Mali [17]. The strains were isolated by direct culture of the black grains obtained by a deep surgical biopsy and were identified at the species level by morphology, polymerase chain reaction with *M*. *mycetomatis* specific primers [18] and sequencing of the internal transcribed spacers [19, 20]. The isolates were maintained in the laboratory on Sabouraud Dextrose Agar (Difco laboratories, Becton and Dickinson, Sparks, USA).

### Screening the Pathogen and Stasis Boxes

In order to screen the Pathogen and Stasis Boxes, an *M*. *mycetomatis* hyphal suspension was made as described previously [21]. In short, *M*. *mycetomatis* was cultured for 7 days at 37°C in RPMI 1640 medium supplemented with 0.3 g/L L-glutamine and 20 mM morpholinepropanesulfonic acid (MOPS). The mycelia were harvested by centrifugation and homogenized by sonication for 20 s at 28 μm (Soniprep, Beun de Ronde, The Netherlands). The fungal suspension was diluted in RPMI 1640 medium to obtain a transmission of 70% at 660 nm (Novaspec II, Pharmacia Biotech) [22]. In each well of a 96-well microplate, 100 μL of suspension was added. 1 μL of compound was added per well to obtain a final concentration of 100 μM. In each assay, two controls were included. These were a growth control (GC), only exposed to the solvent and a negative control consisting of only culture medium. The microplate was taped to prevent evaporation and incubated for 7 days at 37°C. To facilitate end-point reading, 2,3-bis(2-methoxy-4-nitro-5-sulfophenyl)-5-[(phenylamino)carbonyl]-2H-tetrazolium hydroxide (XTT) was added to a final concentration of 25 μg/well and incubated for 2 h at 37°C, and another 3 h at room temperature [22, 23]. Plates were centrifuged and the extinction (E) of 100 μL of supernatant was measured at 450 nm using the spectrophotometer. Percentage growth was calculated by the following formula: (E_sample_-E_nc_)/(E_gc_-E_nc_)*100%. Each compound was tested in triplicate. A hit-compound was defined as a compound resulting in 80% or more reduction in viable fungal mass [21]. To establish which of these hit-compounds were the most potent in inhibiting *M*. *mycetomatis* growth, the concentration of the compound in μM at which a 50% reduction in growth was obtained (IC50) was determined. In order to do this, all positive compounds were tested at concentrations of 0.098 μM, 0.39 μM, 1.56 μM, 6.25 μM and 25 μM and growth was calculated again with the formula (E_sample_-E_nc_)/(E_gc_-E_nc_)*100%. The calculated growth was plotted against concentration and the IC50 was determined from the resulting graphs by visual reading. IC50s were determined in duplicate and the means plus standard deviations were determined in Excel. Four positive growth controls were included in each plate, consisting of a well in which the fungus was exposed to solvent only. Furthermore, two antifungal agents, namely posaconazole and amphotericin B, were included in the plates as positive controls for growth inhibition.

### Minimal inhibitory concentrations of seven *M*. *mycetomatis* strains

To determine if the most potent compounds identified above were also active against *M*. *mycetomatis* isolates with a different genetic background, seven *M*. *mycetomatis* isolates with different AFLP types were selected. Minimal inhibitory concentrations (MIC) were determined using the same protocol as described above. The MIC was defined as the first concentration at which 80% or more reduction in viable fungal mass was obtained [21]. Concentrations ranged from 0.007 μM to 32 μM.

### Toxicity in *G*. *mellonella* larvae

To assess the toxicity of the compounds identified in the *in vitro* screenings *in vivo*, each compound was tested for toxicity in *G*. *mellonella* larvae. For each compound, a single dose was injected into the last right proleg with an insulin needle. Final concentrations of compound in each larva were 0.2 μM, 2 μM or 20 μM of compound. Controls were injected with distilled water only. Larval survival was monitored for 10 days. A non-significant difference in larval survival between the treated group and the control group indicated a lack of toxicity up to the dose under investigation.

### Infection of *G*. *mellonella* larvae with *M*. *mycetomatis* and antifungal treatment

*G*. *mellonella* larvae were infected with *M*. *mycetomatis* isolate Mm55 according to a previously published protocol [24]. In short, *M*. *mycetomatis* mycelia were cultured in colourless RPMI 1640 medium supplemented with L-glutamine (0.3 g/L), 20 mM morpholinepropanesulfonic acid (MOPS) and chloramphenicol (100 mg/L; Oxoid, Basingstoke, United Kingdom) for 2 weeks at 37°C and then sonicated for 2 min at 28 μm. The resulting homogenous suspension was washed once in PBS and further diluted to an inoculum size of 4 mg wet weight per larva. Inoculation was performed by injecting 40 μL of the fungal suspension into the last left pro-leg with an insulin 29G U-100 needle (BD diagnostics, Sparsk, USA). Larvae injected with PBS were included as controls. Larvae were treated with 20 μM of compound per larva or with solvent only. The amount of compound needed to reach a final concentration of 20 μM of compound per larva was calculated and 25 times concentrated solutions were made. The compound was initially dissolved in DMSO and further diluted in such a manner that each injection consisted of a maximum DMSO concentration of only 5%. Each group consisted of 15 larvae, and each experiment was performed three times. The results of the three individual experiments were pooled and plotted in survival curves using GraphPad Prism 5. To treat the larvae, 20 μl of 25 times concentrated compound was administered at 4, 28 and 52 hours after infection. For each injection a different pro-leg was used. Treatment was started at 4 hours since grains were already visible at that time point. To monitor the course of infection, larvae were checked daily for survival for 10 days. If during these 10 days larvae formed pupa, these individuals were not considered further, since we could not ascertain whether these individual larvae would have survived or died during the course of the infection. This means that within each treatment group, the maximum number of larvae was 45 (if no pupae were formed).

### Fungal burden

To determine the fungal burden, 5 larvae from each group were sacrificed at day 3 post infection. First, haemolymph was collected and measured as described earlier [24] to assess melanization. To assess the number of grains per larva, larvae were fixed in 10% buffered formalin and dissected longitudinally into two halves with a scalpel and processed for histology [25]. Sections were stained with haematoxylin and eosin (HE) and Grocott methanamine silver, and grains were manually counted under a light microscope mounted with a Canon EOS70D camera (Canon Inc) by two independent scientists. As controls, infected and non-infected larvae treated with PBS were used. Grains were magnified 40x and visualized on the computer screen using the supplied EOS Utilitysoftware (Canon Inc). Grains were categorized into large, medium or small sizes using the enlargement display frame present in the Live View Shooting mode. Under 40x magnification, the enlargement display frame has a width and height of approximately 250 μm and 160 μm and sums up to a dimension of 0.04 mm^2^. Grains that were larger than half of the display frame were categorized as large (>0.02 mm^2^). Grains that were larger than a quarter of the frame but smaller than half of the frame are categorized as medium (0.01–0.019 mm^2^) and those between one-eighth to a quarter of the display frame (0.005–0.009 mm^2^) were categorized as small. The sum of all large, medium and small grains present in larvae was used to represent the total number of grains in the larvae. To determine the total size of grains in the larvae, the sum of all grains in a larva multiplied by the minimum size of their respective category (large: 0.02mm^2^, medium: 0.01mm^2^ and small: 0.005mm^2^) was used.

### Statistical analysis

To compare survival curves, the Log-rank test was performed with GraphPad Prism 5 (version 5.03, GraphPad Inc.) To determine if there was a statistical difference across the different groups in terms of melanization and grain counts, the Kruskal-Wallis test was performed with GraphPad Prism 5. If a difference was found with the Kruskal-Wallis test, pair-wise comparisons were made between the PBS treated groups and the different antifungal treated groups with the Mann-Whitney U test with GraphPad Prism 5 to determine differences in melanization or CFU count. A p-value smaller than 0.05 was deemed significant. All negative values after normalization were refigured to zero for statistical analysis. To determine the statistical difference in the total number and sizes of grains between the treated and non-treated groups, a Mann-Whitney test was performed with GraphPad Prism 5. A p-value smaller than 0.05 was deemed significant.

## Results

### *In vitro* screening of the Pathogen and Stasis Boxes identify MMV006357 and MMV689244 as the most potent exploratory compounds

**Fig 1:**
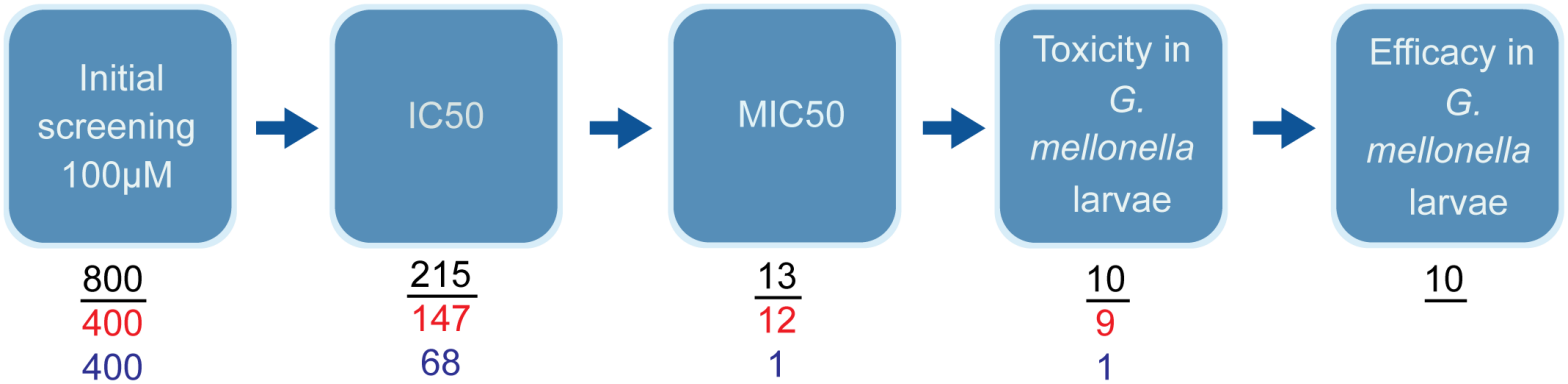
Flow diagram for *in vitro* and *in vivo* evaluation of all compounds (red and blue numbers show numbers of hits from the Pathogen and Stasis Boxes respectively, with the black numbers indicating totals). As is seen in this figure, we started by screening 800 compounds *in vitro* at a concentration of 100 μM. Of those 800 compounds, 400 compounds originated from the Pathogen Box (red) and 400 compounds from the Stasis Box (blue). For the 215 compounds which were inhibited at 100 μM, IC50s were determined. IC50 was defined as the concentration of compound in μM were 50% reduction in fungal growth was obtained. In total, 13 compounds had an IC50 of 5 μM or lower, 12 originating from the Pathogen Box and 1 from the Stasis Box. Of these 13 compounds, MICs were determined against 7 clinical *M*. *mycetomatis* isolates. The MIC was defined as the first concentration at which 80% or more reduction in viable fungal mass was obtained. For the 10 most potent compounds, toxicity and efficacy in *G*. *mellonella* larvae was determined.

**Fig 2:**
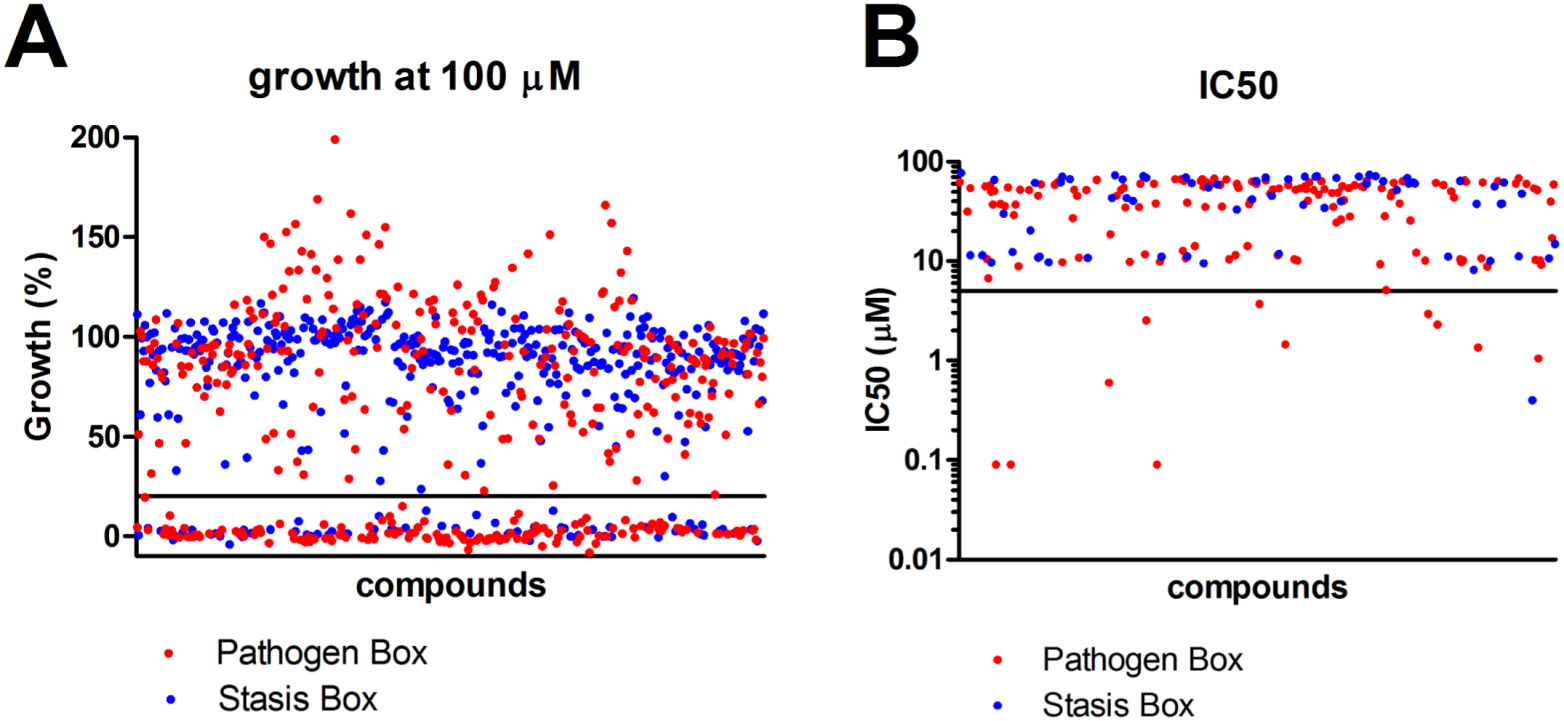
Percentage growth at 100 μM (panel A) and IC50 (panel B) of selected compounds. A. The percentage growth at 100 μM was determined by the XTT assay. Percentage growth was calculated using the following formula: (E_sample_-E_nc_)/(E_gc_-E_nc_)*100%. Percentage growth for each compound in the Pathogen Box is indicated with a red dot, and in the Stasis Box with a blue dot. Compounds with a growth reduction of more than 80% and thus a growth below 20% (black line) were considered compounds able to inhibit *M*. *mycetomatis* growth. Of these compounds the IC50 was determined. B. The IC50 was defined as the concentration of compound in μM were 50% reduction in fungal growth was obtained. The obtained IC50 for the 215 compounds analysed were indicated with a red dot (Pathogen Box) or a blue dot (Stasis Box). Compounds with IC50s below 5 μM (black line) were the most potent compounds which were analysed further.

**Fig 3:**
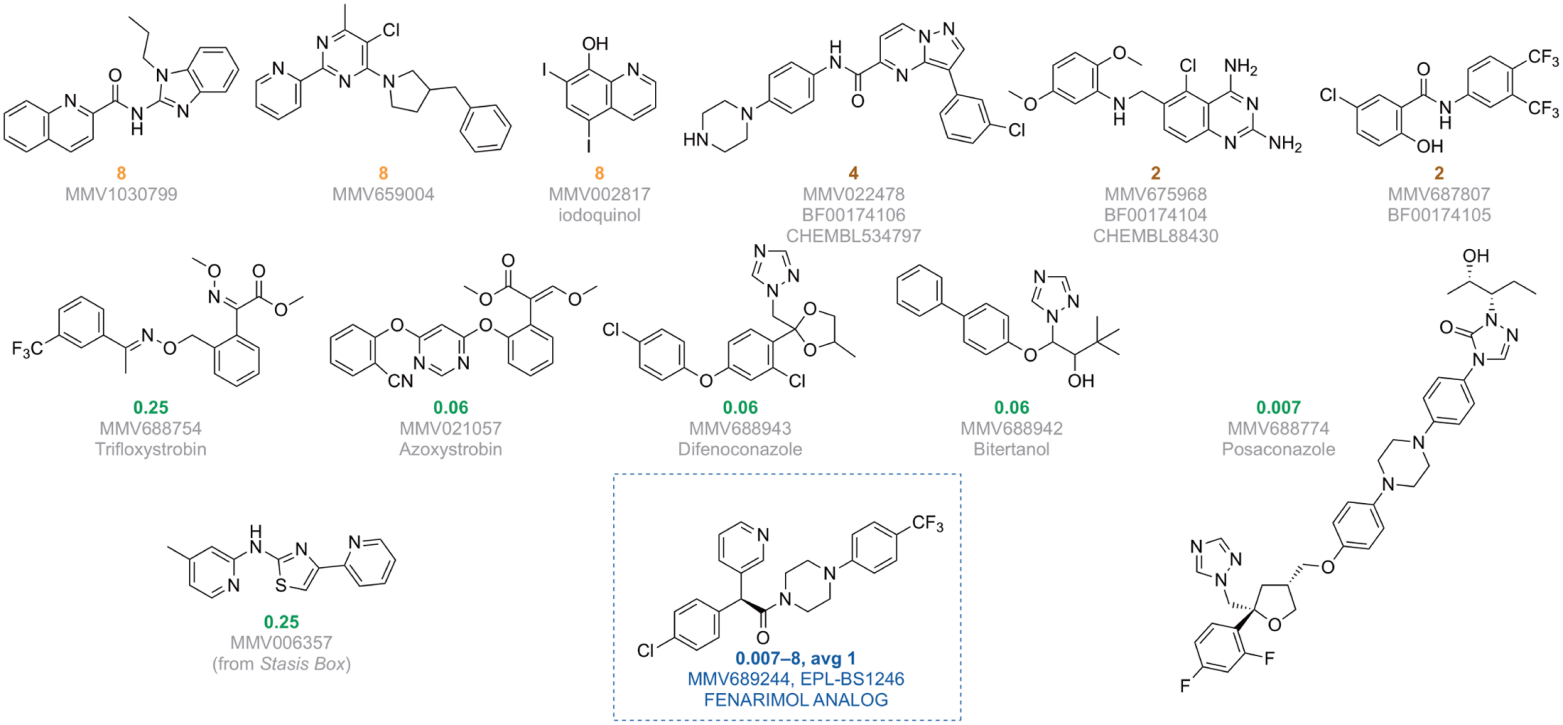
MIC50 in micromolar obtained after testing each of the compounds against seven different *M*. *mycetomatis* isolates. In this figure the compounds are depicted for which the MIC50s were determined. The MIC50 was defined as the concentration for which 50% of tested isolates were completely inhibited in growth. The MIC50 in μM is depicted in orange, brown or green dependent on their activity. The lowest MIC50 was obtaiend for posaconazole, the highest for MMV1030799, MMV659004 and MMV002817. In the blue box, the MIC50 of MMV689244, fenarimol analogue EPL-BS1246 is depicted. For this compound, its MIC ranged from 0.007 to 8 μM, with a MIC50 of 1 μM. Orange MIC50 values indicate low potency, brown MIC50 values indicate intermediate potency and green (<2 μM) indicates high potency.

In total, 800 different drug-like compounds originating from either the Pathogen or Stasis Boxes were evaluated for their potential to inhibit the growth of *M*. *mycetomatis in vitro* and *in vivo* using a sequential assay workflow (Fig 1). Of the 800 compounds screened at a concentration of 100 μM, 215 compounds inhibited growth of *M*. *mycetomatis*. The inhibition of growth was defined as a 80% reduction in fungal growth (<20% growth). Of these 215 compounds, 147 originated from the Pathogen Box and 68 from the Stasis Box (Figs 1 and 2A and S1 Table). A significantly higher hit rate was obtained with the Pathogen Box (36.8%) than with the Stasis Box (17.0%) (Fisher Exact, p<0.0001). To determine which of these compounds were most potent at inhibiting the growth of *M*. *mycetomatis*, the IC50 values of these 215 compounds were determined (S1 Table, Fig 2B). The IC50 was determined by plotting the growth percentage at fixed concentrations and determining the concentration at which 50% reduction of growth was obtained. It appeared that the median IC50 of these 215 compounds was 47.8 μM (<0.09–77.5). In total, 13 compounds had an IC50 of 5 μM or lower (Table 1). Twelve compounds originated from the Pathogen Box and one from the Stasis Box. The antifungal agent amphotericin B, also present as a reference compound in the Pathogen Box, had an IC50 of only 9.7 μM and was therefore excluded from further evaluation; this compound had previously been demonstrated to be active against *M*. *mycetomatis* both *in vitro* [21] and *in vivo* [26]. To determine if the 13 most potent compounds were also able to inhibit *M*. *mycetomatis* isolates with a different genetic background or origin, six other *M*. *mycetomatis* isolates were selected based on their AFLP type or origin and MICs were determined for all 13 compounds. The MIC was defined as the first concentration were a 80% reduction in fungal growth was obtained. To determine the MIC50 for the seven tested isolates, the concentration for which at least 50% of isolates was completely inhibited in growth was determined. As can be seen in Table 1, Fig 3 and S1 Table, MMV688774 (posaconazole, a triazole-based drug approved for the treatment of fungal infections in humans) was the most potent inhibitor of *M*. *mycetomatis* growth with an MIC50 of <0.007 μM. Two azoles, MMV688942 (bitertanol) and MMV688943 (difenoconazole), and two strobilurins, MMV021057 (azoxystrobin) and MMV688754 (trifloxystrobin)—all 4 products being used as agrochemicals—also had strong activity against *M*. *mycetomatis* with MIC50s ranging from 0.06 μM to 0.125 μM. The remaining 8 hits—all part of the non-reference set of exploratory compounds—displayed lower potencies against *M*. *mycetomatis* with MIC50 values ranging from 0.25 μM to 8 μM. Compounds MMV006357 and MMV689244 were the most potent of these 8, displaying MIC50 values of 0.25 μM and 1 μM, respectively. Of note, compound MMV689244 is a fenarimol analogue, identified as a potent inhibitor of *Trypsonosoma cruzi* during a targeted screening exercise for new drugs for Chagas disease [27–29], during which over 800 fenarimol analogues were synthesized (MMV689244 corresponds to EPL-BS1246 in this library) and addressing, through chemical modification including scaffold-hopping, the few identified liabilities of the fenarimol series notably low solubility, high *in vitro* metabolism and inhibition of cytochrome P450 3A4/5 (CYP3A4/5). This work resulted in the identification of a couple of optimized leads associated with a CYP51 selective profile (i.e. lacking cytochrome P450 3A4 inhibition) with nanomolar *in vitro* potency *vs*. *T*. *cruzi*, excellent *in vivo* efficacy in an infected murine model [27] and a lack of adverse events on extended dosing in murine exploratory toxicity studies.

**Table 1:** IC50 and MIC50 against *M*. *mycetomatis* of the 13 most active compounds identified in the Pathogen and Stasis Boxes.

	Trivial name or CHEMBL code	Class	MoA	use	IC50 of strain MM55	MIC50
**MMV688774**	Posaconazole	Azoles	CYP51 inhibitor	antifungal (human)	<0.10	<0.007
**MMV688942**	Bitertanol	Azoles	CYP51 inhibitor	antifungal (agrochemical)	<0.10	0.06
**MMV688943**	Difenoconazol	Azoles	CYP51 inhibitor	antifungal (agrochemical)	<0.10	0.06
**MMV021057**	Azoxystrobin	Strobilurins	Complex III mitochondrial electron transport chain binder	antifungal (agrochemical)	0.60	0.06
**MMV688754**	Trifloxystrobin	Strobilurins	Complex III mitochondrial electron transport chain binder	antifungal (agrochemical)	1.05	0.25
**MMV006357**	N/A	2-aminothiazole	N/A	N/A	0.40	0.25
**MMV689244**	EPL-BS1246	Fenarimols (non-azoles)	CYP51 inhibitor	antiprotozoal (Chagas disease preclinical candidate)	1.35	1
**MMV687807**	N/A	Benzamides	N/A	N/A	1.45	2
**MMV675968**	CHEMBL88430	Quinazolines	Dihydrofolate reductase	N/A	2.30	2
**MMV022478**	CHEMBL534797	Pyrazolo-pyrimidines	N/A	N/A	2.95	4
**MMV002817**	Iodoquinol	Quinoline	Unknown	antiprotozoal (amoebiasis)	2.55	8
**MMV1030799**	N/A	Benzimidazole quinoline	N/A	N/A	3.70	8
**MMV659004**	N/A	Pyridyl-pyrimidine	N/A	N/A	5.10	8

### *In vitro* screening of the 35 MMV689244 analogues identifies four potent fenarimol analogues

**Fig 4:**
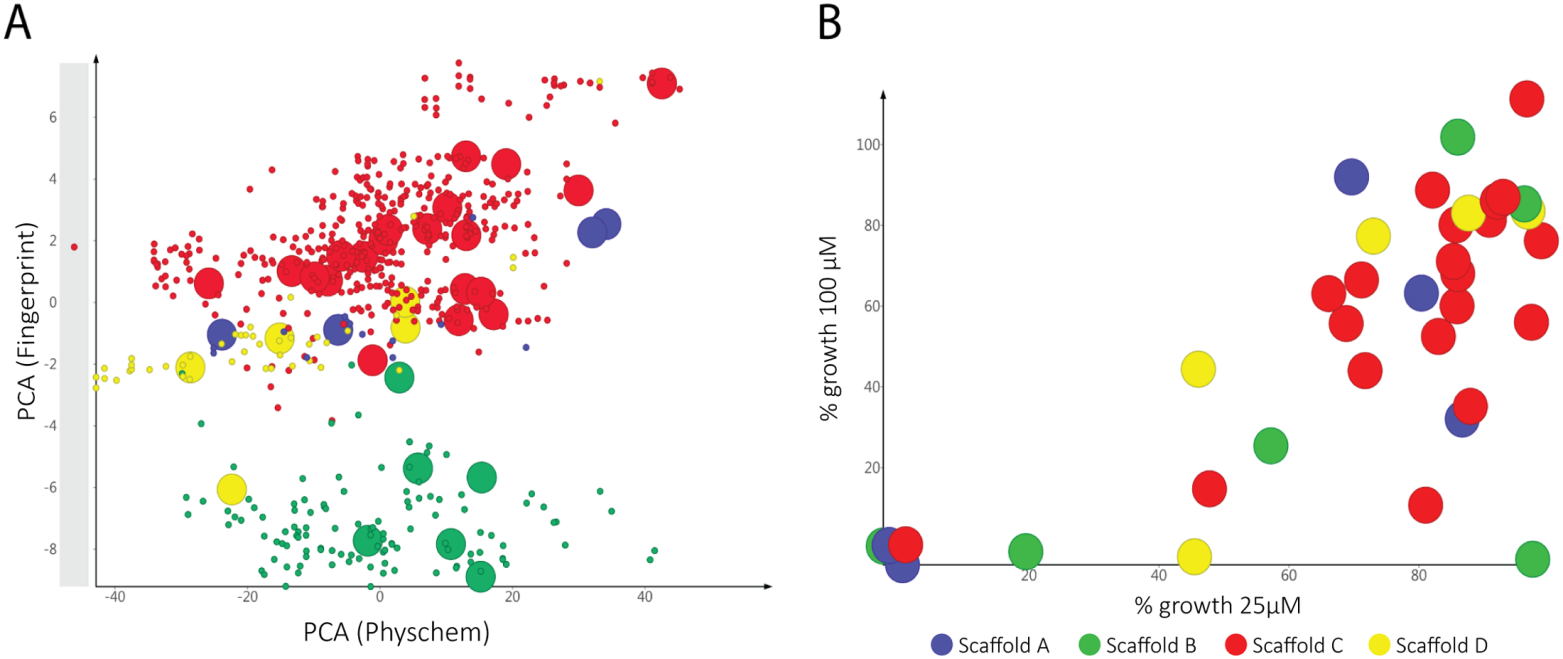
A) 2-D Chemical space representation of the 800 member fenarimol analogue library. X -axis represents a Principal Component Analysis of approximately 100 different physichochemical properties, Y-axis represents a Principal Component Analysis of 1024 Morgan chemical fingerprints (all calculations performed using RDKit in KNIME)[52] Compounds chosen for test are represented by oversized points, and different core scaffolds represented by colour (Blue -scaffold A; green scaffold B, red scaffold C, yellow scaffold D). B: % Growth inhibition by selected fenarimol analogues at 25 and 100 μM. Percentage growth was calculated using the following formula: (E_sample_-E_nc_)/(E_gc_-E_nc_)*100%.

**Fig 5:**
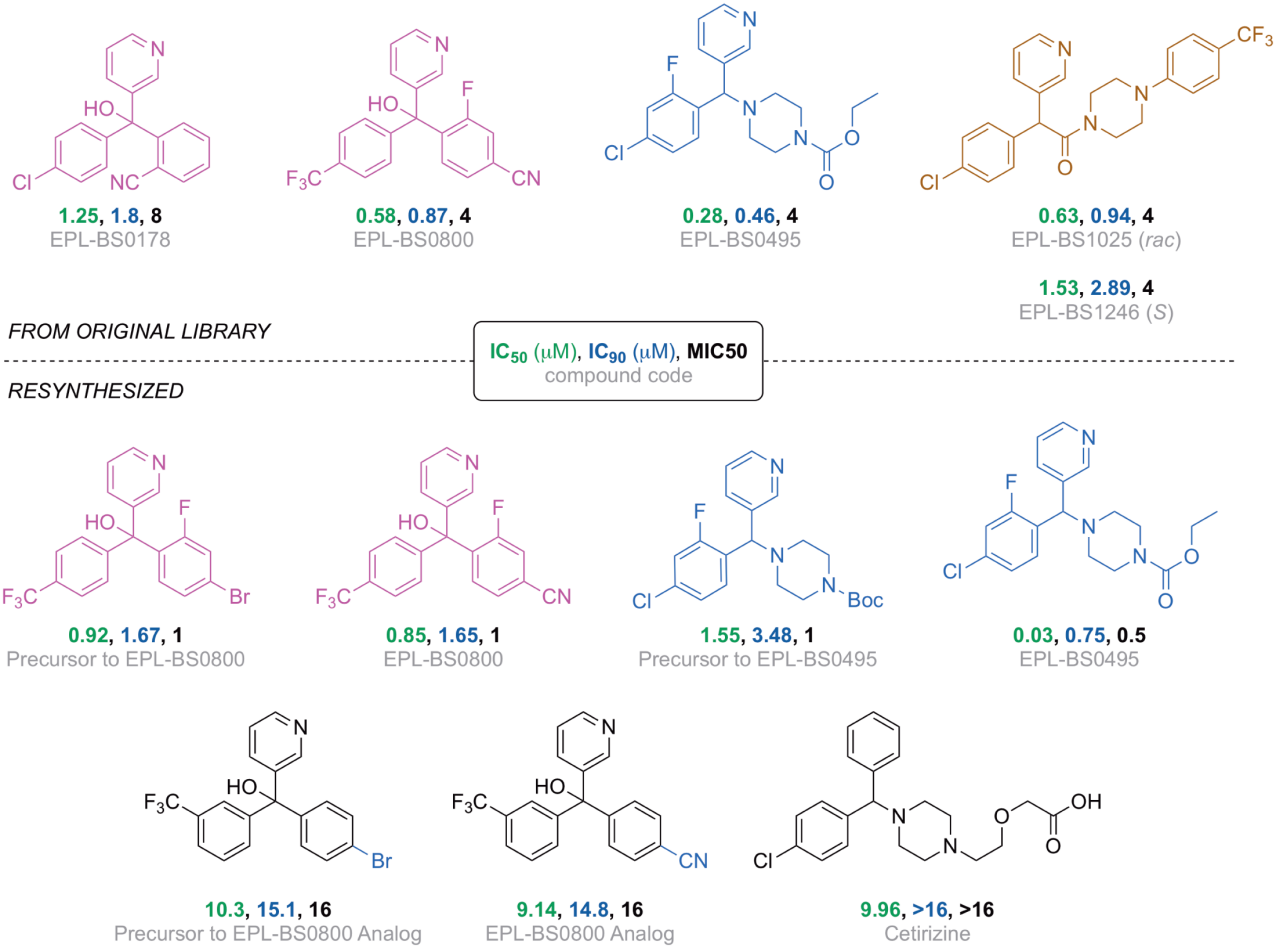
*In vitro* potency of the active compounds from the fenarimol library. Originally-evaluated samples (top); resynthesized samples and analogs (bottom). In this figure, the IC50, IC90 and MIC50 are depicted in green, blue and black. The compound code is depicted in gray.

Due to the potent inhibition of *M*. *mycetomatis* by MMV689244, as well as the availability of the compound collection to our screening program, an additional 35 diverse compounds from the original 800 fenarimol analogue set were tested to determine their *in vitro* activity against *M*. *mycetomatis* hyphae (S1 Table). These compounds were selected based on similarity to the original hit whilst ensuring a diverse sampling of the various scaffold variation within the chemical set. Analogues tested included variations of substituents around the fenarimol core as well as compounds with closely related scaffolds, since MMV689244 itself is a scaffold variant of fenarimol. The diversity of this selection of compounds for screening was confirmed by chemical space analysis using principal component analysis of both physicochemical and chemical fingerprint evaluation of the library (Fig 4). Fenarimol analogues were tested for potential inhibition of the growth of *M*. *mycetomatis* at both 100 and 25 μM and the most potent compounds were then subjected to dose response determinations. Four of the analogues demonstrated inhibition of growth at both concentrations (Fig 5, top): EPL-BS0495, EPL-BS0800, EPL-BS1025 (racemate of EPL-BS1246) (all having MIC50 values of 4 μM) and EPL-BS0178 (MIC50 of 8 μM) (Fig 5). Interestingly, the potency of these analogues was not confined to a single scaffold type, with different representatives of the fenarimol core and fenarimol inspired scaffold changes resulting in MIC50 values in the micromolar (2–8 μM) range (Fig 5 and S1 Text). In addition, EPL-BS1025 demonstrated similar potency to its enantiopure counterpart from the original assay (EPL-BS1246, MMV689244), validating this hit from the original screen. Analysis of the data around these 35 selected fenarimol analogues revealed few conclusive structure activity relationship (SAR) observations. We attribute this to the diversity of the chemical space scanned, and further focused screening of the available analogue chemical space is expected to reveal key SAR.

Resynthesis of the fenarimol analogs was achieved using methods largely based on procedures found in the literature [27–29] as described in the supporting information (S2 Text), and these compounds were validated as hits (Fig 5, bottom). The resynthesized EPL-BS0800 and EPL-BS0495 showed potent *in vitro* activity, with a slightly higher IC50 for the resynthesized EPL-BS0800 (0.85 vs 0.58) and a slightly higher IC50 for the resynthesized EPL-BS0495 (0.03 vs 0.28) compared to the original compounds. Several synthetic precursors and a novel analog synthesized with minor changes in the pendant rings of EPL-BS0800 were also evaluated. While the novel analog of EPL-BS0800 did not provide *in vitro* potency, the precursors to EPL-BS0800 itself (containing a bromine atom in place of a cyano group) and to EPL-BS0495 (containing a Boc-protecting group rather than the ethyl carbamate) were active. These results suggest that either the carbamate group in EPL-BS0495 is labile under the assay conditions or there is room for tolerance to variation in this position on the molecule. The commercially available anti-inflammatory compound cetirizine (Zyrtec), which possesses striking structural similarity to the fenarimol analogs, was evaluated but found to be inactive.

### *In vivo* tolerability of the 10 most potent compounds identified from the Pathogen and Stasis Boxes

The 10 most potent hits resulting from screening the Pathogen and Stasis Boxes were further evaluated *in vivo* using the *G*. *mellonella* larvae model. In this model, mycetoma grains are produced which resemble the grains present in human patients or mammalian models [24]. Furthermore, the therapeutic efficacy obtained in this model with standard antifungal agents [30], was comparable to that obtained in a mouse model [31]. Therefore this model was considered a good screening model to assess *in vivo* activity of the hit compounds against *M*. *mycetomatis* grains. A first requirement was to determine if these compounds displayed any toxicity to the larvae. This was assessed by injecting a single dose of compound into the hemolymph of the larvae. Survival was monitored for 10 days. At the highest concentration tested (20 μM/larvae), none of the compounds displayed toxicity.

### *In vivo* activity of the 10 most potent compounds identified from the Pathogen and Stasis Boxes

**Fig 6:**
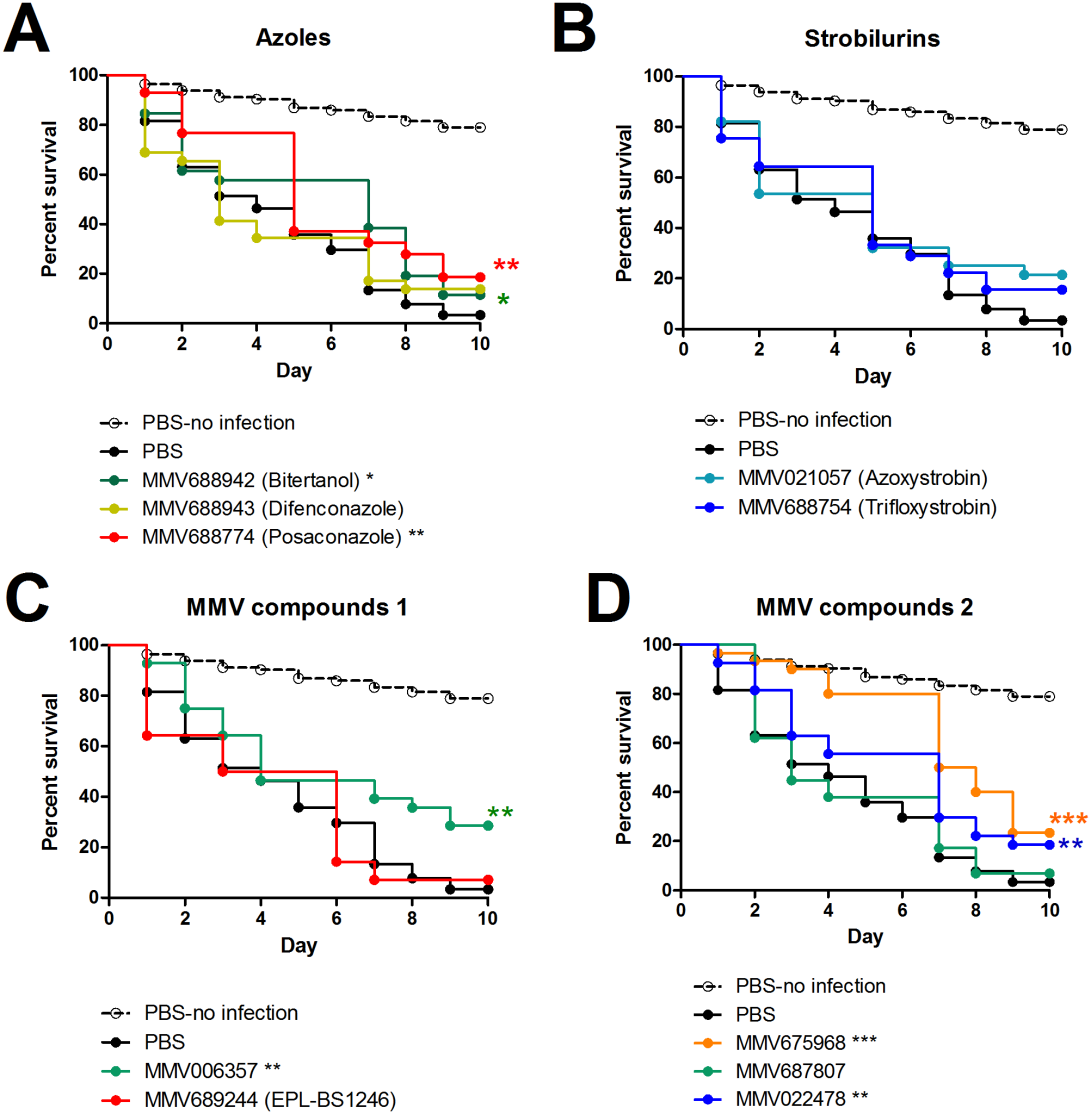
Survival curves of larvae infected with *M*. *mycetomatis* and treated with selected compounds. The black dashed line in all panels correspond to larvae mock-inoculated with PBS and treated with PBS. The black line in all panels corresponds to *M*. *mycetomatis* infected larvae treated with PBS. These are the control lines. In panel A, the survival of larvae treated with azoles MMV688942 (Bitertanol), MMV68893 (Difenconazole) and MMV688774 (Posaconazole) is displayed. In panel B, the survival of larvae treated with strobilurins MMV021057 (azoxystrobin) and MMV688754 (trifloxystrobin) is displayed. In panels C and D, the survival of larvae treated with the MMV compounds is displayed. These include MMV006357, MMV6894244 (EPL-BS1246), MMV675968, MMV687807 and MMV022478. Significant survival was displayed as * (0.01<p<0.05), ** (0.001<p<0.01) or *** (0.001<p).

Since none of the compounds were considered toxic (S3 Text), therapeutic efficacy was determined in *M*. *mycetomatis* infected larvae. Of the reference compounds used, only MMV688774 (posaconazole) (Log-Rank, p = 0.011) and MMV688942 (bitertanol) (Log-Rank, p = 0.0178) prolonged survival in this model in a statistically significant manner (Fig 6A). MMV688943 (difenconazole), MMV021057 (azoxystrobin) and MMV688754 (trifloxystrobin) did not prolong larval survival (Fig 6A and 6B). Of the five other compounds tested, only MMV006357 (Log-Rank, p = 0.0012), MMV675968 (p<0.0001) and MMV022478 (p = 0.0224) prolonged survival in a statistically significant manner (Fig 6C and 6D). The highest overall survival was obtained with compound MMV006357, which resulted in an overall survival of 28.6%. Compounds MMV675968 and MMV022478 demonstrated a lower overall survival percentage, but prolonged survival more effectively at the beginning of the infection.

**Fig 7:**
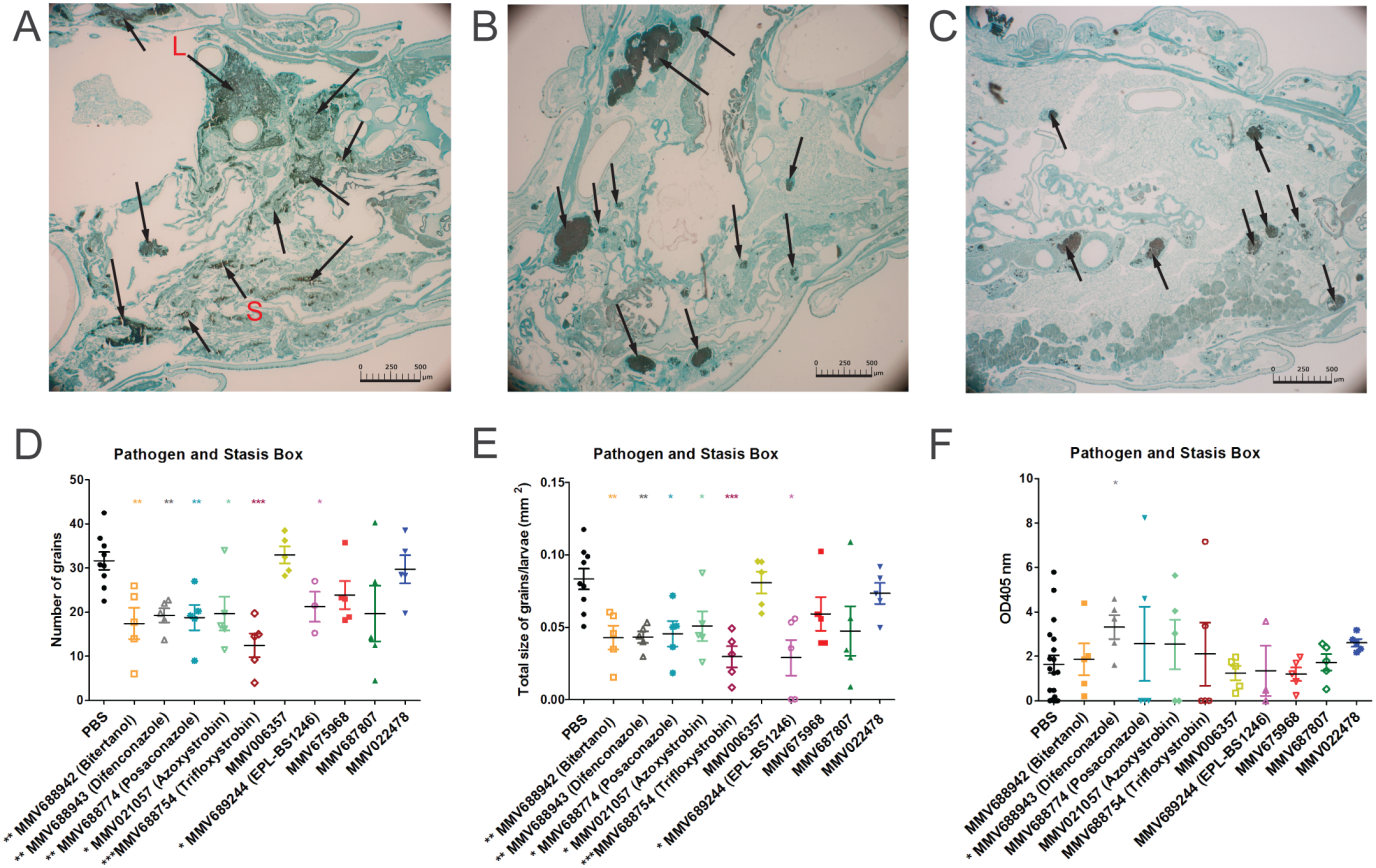
Fungal burden in *G*. *mellonella* larvae infected with *M*. *mycetomatis* and treated with selected compounds. In panels A, B and C, histopatholocial sections of larvae are demonstrated which were treated with the different compounds and sacrificed 72h after inoculation. Histophatological sections are stained with Grocott to demonstrate the presence of fungal grains (black stained) and indicated by arrows. Panel A, demonstrate a larvae treated with PBS as a control, for which both large grains (L) and smaller grains (S) are visible. Panels B and C show *G*. *mellonella* infected with *M*. *mycetomatis* and treated with MMV688942 (Bitertanol)(B), MMV689244 (EPL-BS1246)(C). The scale bar present on each image represents 500μm. By counting the grains on these histological sections of 3–5 larvae per group, the number of grains (panel D) and the total grain size (panel E) per larvae per treated compound or PBS (control group) was determined. Melanisation of the larvae was determined by measuring the OD405nm of the hemolymph in all groups (panel F). PBS in all panels corresponds to *M*. *mycetomatis* infected larvae, treated with PBS. This is the control group. Significant differences determined using the Mann-Whitney U-test were displayed as * (0.01<p<0.05), ** (0.001<p<0.01), or *** (p<0.001).

To determine if the compounds had any effect on the fungal burden 5 larvae from each group were sacrificed at day 3 post infection and histology was performed. The total number of grains per larvae and the size of the individual grains were assessed. As can be seen in Fig 7A, in PBS treated larvae a large number of grains can be seen and they vary in size ranging from very large (L) to small (S). Grain appearance differed when larvae were treated with either MMV688942 (bitertanol) (Fig 7B) or MMV689244 (EPL-BS1246) (Fig 7C). Both number and size of grains appeared smaller. When the total number of grains (Fig 7D) and size of the grains (Fig 7E) per group of larvae was assessed it appeared that there was both a significant reduction in the number of grains and total size of grains per larvae when larvae were treated with MMV688942 (bitertanol), MMV688943 (difenconazole), MMV688774 (posaconazole), MMV021057 (azoxystrobin), MMV688754 (trifloxystrobin)and MMV689244 (EPL-BS1246) (Fig 7D and 7E). No difference in the distribution of large, medium and small grains was noted for any of the groups, except for MMV688942 (bitertanol) treated larvae (Table 2). In that group, a significantly lower number of large grains and a higher number of smaller grains was noted compared to the PBS treated larvae. Since for certain compounds a lower fungal burden was obtained, it was hypothesized that this would also reflect in a difference in the immune reaction of the larvae towards the fungi. Melanization of the haemolymph is part of the immune system of *G*. *mellonella* larvae and it indicates the extent of the immune response elucidated towards a pathogen. Therefore the melanisation of the hemolymph was measured in the larvae (Fig 7F). A significant decrease in melanization was observed in MMV688943 (difenconazole) (Mann-Whitney, p = 0.047) treated larvae, whereas no significant difference in melanization was observed with other compounds (Fig 7F).

**Table 2:** Statistical analysis of the total number of grains in larvae treated with the 14 compounds.

Compounds	*n*	Median			Median of total grain number	Total number			p-value (Chi-square)
		Large n(STDEV)	Medium n(STDEV)	Small n(STDEV)		Large	Medium	Small	
**Control**									
PBS	9	2.8 (2.1)	8.5 (3.2)	21.8 (3.6)	31	32	68	185	-
**Pathogen and Stasis Box**									
MMV688942 (Bitertanol)	5	1.5 (0.8)	3 (1.6)	12.5 (6.0)	18	8	17	62	0.547
MMV688943 (Difenconazole) *	5	1.5 (0.9)	2.8 (0.9)	15.5 (3.2)	20	6	15	76	0.047 *
MMV688774 (Posaconazole)	5	1.8 (1.5)	3.3 (1.9)	13.5 (4.5)	19	9	17	69	0.373
MMV021057 (Azoxystrobin)	5	1.8 (1.3)	4.5 (1.5)	10.3 (6.1)	17	10	23	65	0.953
MMV688754 (Trifloxystrobin)	5	0.3 (1.3)	2.3 (1.2)	10.3 (4.4)	15	5	11	46	0.373
MMV006357	5	3 (2)	6 (1.8)	22.5 (2.8)	33	15	33	117	0.425
MMV689244 (EPL-BS1246)	3	1.3 (1)	4.3 (1.5)	14 (5.7)	22	4	12	48	0.266
MMV675968	5	2 (2)	4.5 (2.4)	15.8 (2.6)	23	11	26	83	0.689
MMV687807	5	1.2 (1.9)	2.5 (3.7)	10.5 (9.1)	14	8	18	72	0.297
MMV022478	5	3 (0.9)	5.5 (1.2)	21 (5.7)	29	14	29	106	0.423
**Fenarimol analogues**									
EPL-BS0178	5	1.3 (1)	7 (2.5)	15.8 (5.9)	26	7	32	96	0.127
EPL-BS0495	5	2.5 (1.8)	5 (2.1)	20.8 (7.9)	28	16	21	94	0.195
EPL-BS0800	5	1.3 (0.9)	2.5 (1.6)	12 (9.4)	18	5	14	68	0.062
EPL-BS1025	5	2.8 (1.6)	2.5 (4.5)	11.8 (7.3)	17	10	20	60	0.946

*Significant difference is displayed as (0.01<p<0.05)

### *In vivo* activity of the fenarimol analogues

**Fig 8:**
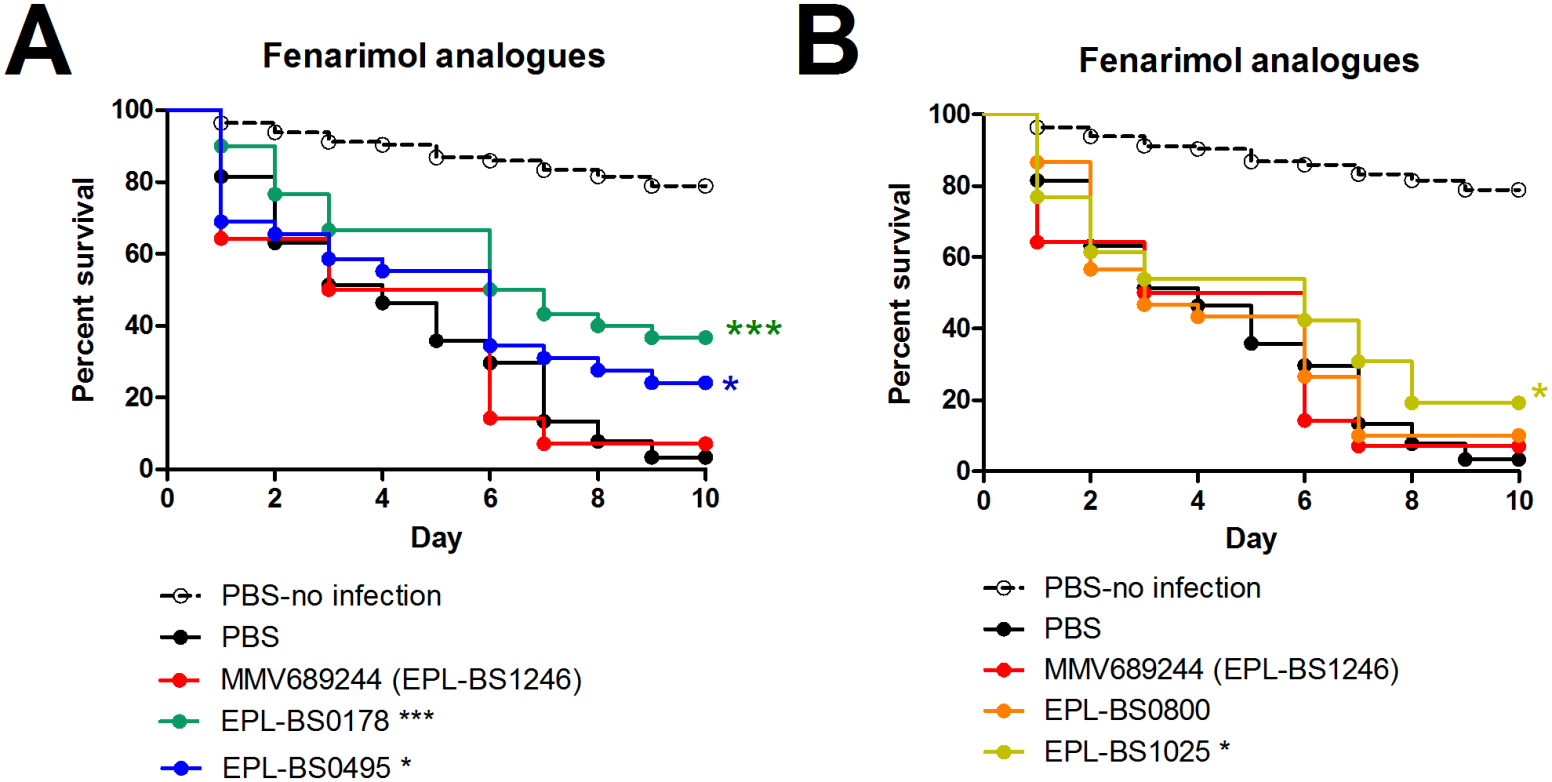
Survival curves of larvae infected with *M*. *mycetomatis* and treated with fenarimol analogues. The black dashed line in all panels correspond to larvae mock-inoculated with PBS and treated with PBS. The black line in all panels corresponds to *M*. *mycetomatis* infected larvae treated with PBS. These are the control lines. In panels A and B, the fenarimol analogues MMV689244 (EPL-BS1246), EPL-BS0178, EPL-BS0495, EPL-BS0800 and EPL-BS1025 are displayed. Significant survival was displayed as * (0.01<p<0.05), ** (0.001<p<0.01) or *** (0.001<p).

**Fig 9:**
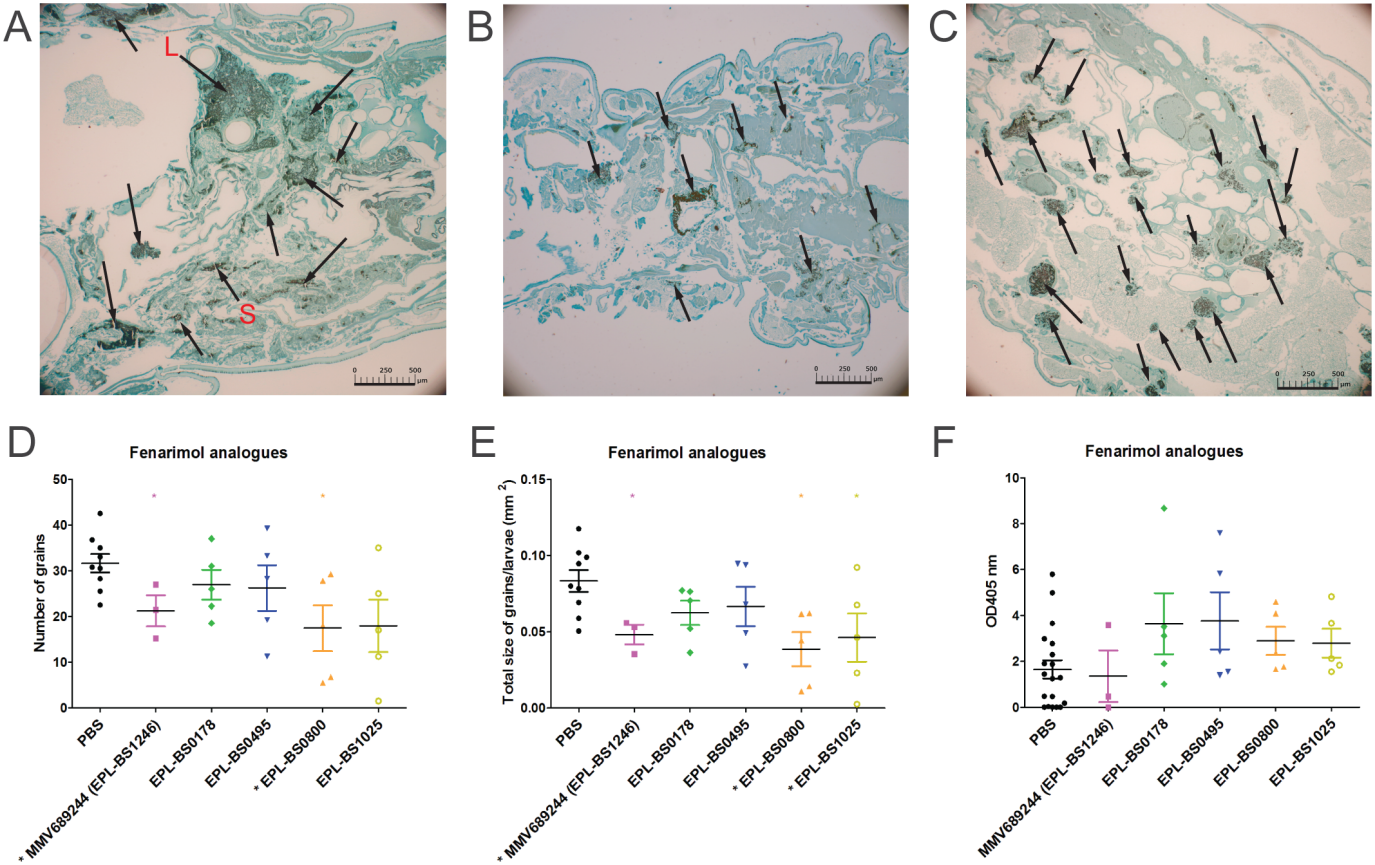
Fungal burden in *G*. *mellonella* larvae infected with *M*. *mycetomatis* and treated with fenarimol analogues. In panels A, B and C, histopatholocial sections of larvae are demonstrated which were treated with the different compounds and sacrificed 72h after inoculation. Histophatological sections are stained with Grocott to demonstrate the presence of fungal grains (black stained) and indicated by arrows. Panel A, demonstrate a larvae treated with PBS as a control, for which both large grains (L) and smaller grains (S) are visible. Panels B and C show *G*. *mellonella* infected with *M*. *mycetomatis* and treated with EPL-BS0178 (B) and EPL-BS0495(C). The scale bar present on each image represents 500μm. By counting the grains on these histological sections of 3–5 larvae per group, the number of grains (panel D) and the total grain size (panel E) per larvae per treated fenarimol analogue or PBS (control group) was determined. Melanisation of the larvae was determined by measuring the OD405nm of the hemolymph in all groups (panel F). PBS in all panels corresponds to *M*. *mycetomatis* infected larvae, treated with PBS. This is the control group. Significant differences determined using the Mann-Whitney U-test were displayed as * (0.01<p<0.05), ** (0.001<p<0.01), or *** (p<0.001).

One of the five non-reference compounds, MMV689244 (EPL-BS1246), did not prolong larval survival (Fig 8), although it was able to reduce the fungal burden (Fig 9D and 9E). The lack of prolonged survival for MMV689244 (EPL-BS1246) was surprising since it was the compound with the most potent *in vitro* activity after the reference compounds. Although overall survival was not prolonged, a reduction in grain number and grain size was obtained with MMV689244 (EPL-BS1246). Since *M*. *mycetomatis* forms grains *in vivo* but not *in vitro*, we therefore hypothesized that compound MMV689244 (EPL-BS1246) might not be able to reach its target in the grain. We therefore tested the four fenarimol analogues with *in vitro* activity to determine if any would have activity against *M*. *mycetomatis* grains *in vivo*. It appeared that EPL-BS0178 (Log Rank p<0.0001), EPL-BS0495 (Log Rank, p = 0.0199) and EPL-BS1025 (Log Rank, p = 0.0436) were all able to prolong larval survival in a statistically significant manner (Fig 8A and 8B). The overall survival percentages of the three active fenarimol analogues were compared; treatment with analogue EPL-BS0178 resulted in a survival percentage of 36.7%, which was higher than that observed with analogues EPL-BS0495 (24.1%) and EPL-BS1025 (19.2%). Since EPL-BS1025 is the racemate of EPL-BS1246, it is surprising that EPL-BS1025 prolonged larval survival compared to PBS treated larvae whereas MMV689244 (EPL-BS1246) did not. However, when survival curves of *M*. *mycetomatis* infected larvae treated with MMV689244 (EPL-BS1246) were compared to those treated with EPL-BS1025, no significant difference in larval survival was noted (Log Rank, p = 0.21) (Fig 8A and 8B).

The effect of the fenarimol analogues on the fungal burden was determined on 5 larvae from each group at day 3 post infection. When compared to PBS treated larvae (Fig 9A), EPL-BS0178 (Fig 9B) and EPL-BS0495 (Fig 9C) treated larvae had smaller grains. When the complete treatment groups were compared it appeared that only fenarimol analogues MMV689244 (EPL-BS1246) and EPL-BS0800 (median = 18 grains; Mann-Whitney, p = 0.019 and median = 0.044mm^2^; Mann-Whitney, p = 0.012) significantly lowered the total number of grains and the total grain size (Fig 9D and 9E), the two analogues which did not demonstrate enhanced larval survival. EPL-BS0178, EPL-BS0495 and EPL-BS1025 did not significantly lower the number of grains present in the infected larvae (Table 2, Fig 9D). For EPL-BS1025, a significant reduction in total grain size produced was noted (Fig 9E; median mass: 0.046 mm^2^; Mann-Whitney, p = 0.042).No difference in grain distribution (Table 2) or larvae melanization was observed for any of the fenarimol analogues (Fig 9F).

**Fig 10:**
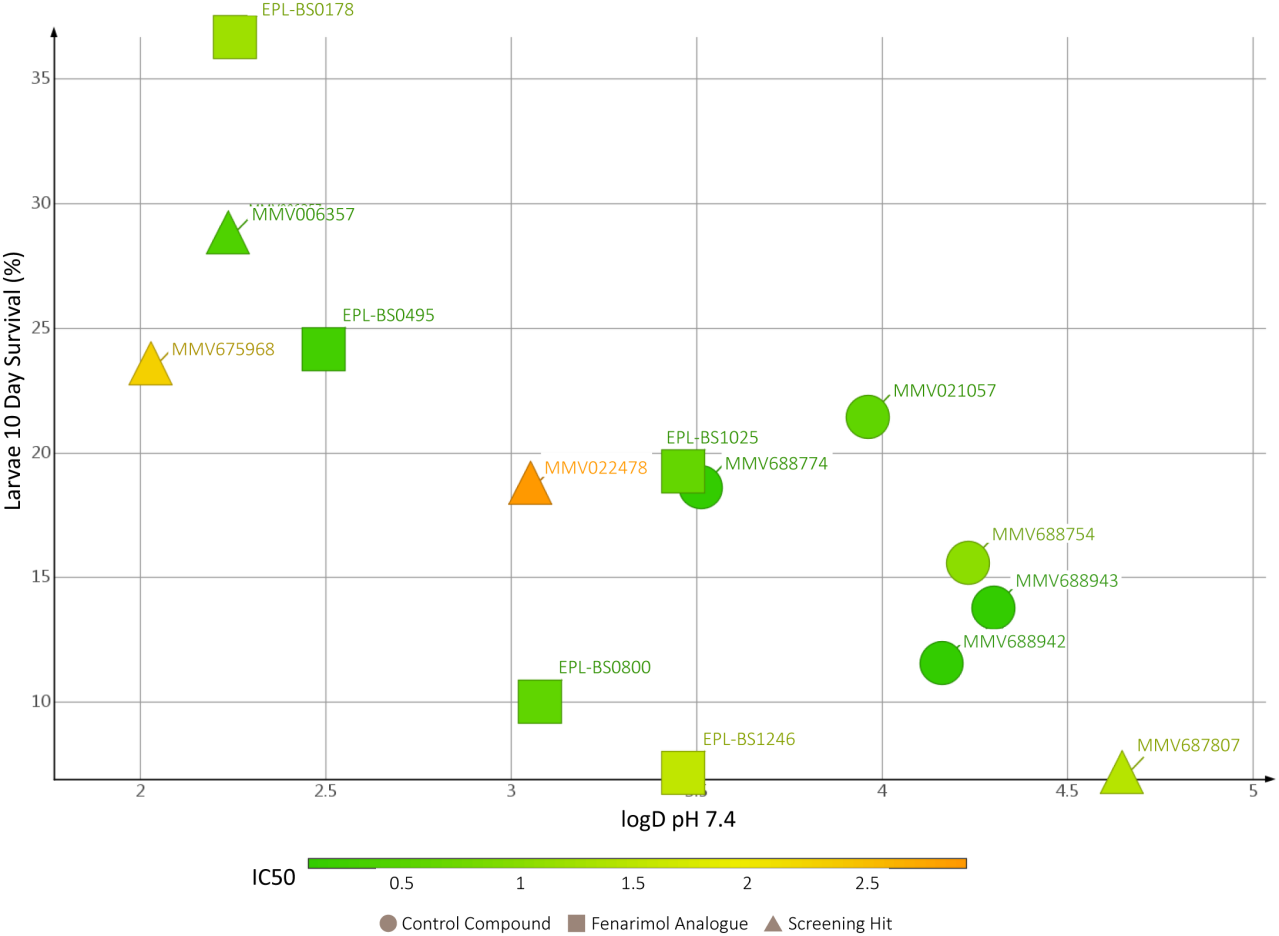
logD pH 7.4 versus *in vivo* larvae survival after 10 days.

To better understand the translation from *in vitro* activity to *in vivo* efficacy, physicochemical profiling of the ten compounds was performed. A correlation between the LogD at pH 7.4 (calculated using Stardrop [version 2017, Optibrium Ltd]), IC50 *in vitro* and percentage survival after 10 days was noted (Fig 10).

## Discussion

In this study, the Pathogen and Stasis Boxes were screened to identify compounds with *in vitro* antifungal activity against *Madurella mycetomatis*. Out of the 800 compounds tested, 13 compounds were associated with *in vitro* activity (determined as MIC50s) against *M*. *mycetomatis* strain Mm55 ranging from nanomolar (<0.007 μM) to micromolar (8 μM) potencies (S1 Table, Table 1, Fig 3). Of these 13 compounds, five are antifungal agents belonging either to the azoles (posaconazole, bitertanol and difenoconazole) or the strobilurins (azoxystrobin and trifloxystrobin), two are antiprotozoal agents (iodoquinol being an approved drug for the treatment of amoebiasis and MMV689244 a preclinical candidate for the treatment of Chagas disease) while the other five compounds had not previously been associated with any antifungal properties, being upstream exploratory molecules. Interestingly, when the Pathogen Box was screened with planktonic cells of other fungal species, such as *Candida albicans* [32] and *Cryptococcus neoformans* [32] MMV688934 (tolfenpyrad) and MMV688271 were also identified as antifungal agents [32], yet in our study MMV688934 did not inhibit *M*. *mycetomatis* growth at all, and MMV688271 only did so with an IC50 of 61.7 μM. In the same study, only six compounds were found to be able to inhibit *C*. *albicans* biofilm formation [33]. Among these compounds, amphotericin B, MMV687807, MMV687273 and MMV688768 were identified [33]. In our assay, amphotericin B had an IC50 of 9.7 μM and was therefore excluded from further evaluation, while MMV687807 was one of the 10 compounds with the highest *in vitro* activity.

To assess the *in vivo* activity of the selected compounds we made use of the previously established *G*. *mellonella* grain model [24]. This model was chosen as it produces grains which resemble grains found in human and it has been demonstrated to predict the *in vivo* activity of antifungal agents in mammalian models. Itraconazole and amphotericin B were evaluated for activity in both *G*. *mellonella* and mouse models of *M*. *mycetomatis* mycetoma. It appeared that at human pharmacokinetic equivalent dosages the itraconazole did not prolong larval survival but amphotericin B did [30]. In mice, amphotericin B was able to prevent grain formation, but itraconazole was not [31]. Therefore, the *G*. *mellonella* model was found to be a good indicator for *in vivo* activity in mammalian models. In the current study, we determined the *in vivo* activity of 10 of the most promising compounds obtained from the Pathogen and Stasis Boxes, five showed enhanced survival with the Log-Rank test (Fig 6). Of these compounds, only posaconazole had previously been tested in this larval model; larvae were treated with 5.7 mg/kg posaconazole, a dose similar to the one used in the treatment of human fungal infections, but this did not lead to any enhancement of larval survival [26]. In the current study, enhanced survival was noted when larvae were treated with 20 μM/larva which is comparable to 14 mg/kg of posaconazole.

The compounds with the most potent *in vivo* activity were MMV006357, MMV675968 and MMV022478. MMV006357 was obtained from the Stasis Box and is a 2-aminothiazole derivative that was originally identified as a molecule with anti-mycobacterial activity [34, 35]. It has been demonstrated to be active against replicating and non-replicating mycobacteria and even had sterilizing activity against the latter [34], a property possessed by no other class of anti-mycobacterial compound. Furthermore, several of its analogues also had activity against drug-resistant isolates of *M*. *tuberculosis* [36]. MMV675968 is a 2-4-diaminoquinazoline derivative active against *Cryptosporidium parvum* while MMV022478 is a pyrazolopyrimidine previously described as active *in vitro* against the asexual blood stage of *Plasmodium falciparum* as well as against *Plasmodium berghei* sporozoites (https://www.pathogenbox.org/).

By comparing the *in vitro* and *in vivo* activity data, it appears that the compounds with the highest *in vitro* activity are usually not the ones with the best *in vivo* activity. This lack of association has been observed in the past, notably with respect to azoles. *In vitro*, *M*. *mycetomatis* was indeed shown to be most susceptible towards the azoles [21, 37] and less susceptible towards the polyene amphotericin B [37]. However, only amphotericin B [21]was able to prolong *G*. *mellonella* larval survival or able to inhibit grain formation in mice [26] [38]. A lack of translation of activity from *in vitro* to *in vivo* could be due to pharmacodynamic considerations or due to the difference in morphological organization of the fungus inside the host. Azole antifungal agents are known to be more active on quickly dividing and faster growing fungi. *In vitro*, *M*. *mycetomatis* is metabolically active and visible growth is seen within days, while *in vivo*, grains are formed. Their metabolic activity is not currently known, but since no expansion of the grains is noted in tissue sections, it is envisioned that the grain itself is less metabolically active. Although lower metabolic activity within the grain might be one reason, it cannot be the only one. In earlier experiments performed by Murray, it was demonstrated that even 100 μg/mL of the fungicidal drug amphotericin B was not able to prevent *M*. *mycetomatis* growth when freshly isolated *M*. *mycetomatis* grains from infected mice were immersed in melted agar, while 8 μg/mL was enough to inhibit the growth of *M*. *mycetomatis* cultured mycelia [39, 40].

Although the grain itself is a key feature of mycetoma, little is known about its constituents or metabolic activity. In the 1970s some attempts were made by Findlay and Vismer to identify the constituents, but since then little progress has been made [41–43]. Findlay showed that the grain owed its existence and its toughness to a tanning of the structural and inflammatory proteins of the host by a diffusible melanin-type pigment synthesized and secreted by the fungus [41]. When this melanin was isolated and added to our *in vitro* susceptibility assay, MICs for ketoconazole and itraconazole were elevated 32 times [44]. Within the grain, intrahyphal growth is observed [43] and the hyphae themselves are embedded in cement material, which makes it difficult for each drug to reach the metabolically active part of the fungus. Although the exact constituents of this cement material are still unknown, chitin [45] and beta-glucan [46] are known to be involved. These constituents are implicated in the reduced antifungal susceptibility of fungal biofilms [47]. In addition, the grain itself is surrounded by a collagen capsule, which also makes it more difficult for the drug to reach the grain itself [48].

We postulated that the activity against the fungal grain of the hits identified *via in vitro* and *in vivo* screening might be enhanced by chemical modification of the original hit structure. This hypothesis was first validated with respect to hit MMV689244 (EPL-BS1246), one of the most potent *in vitro* hits identified during screening of the Pathogen Box but which did not, however, significantly enhance survival when evaluated in the *G*. *mellonella* larvae model. The Indeed, when four fenarimol analogues with *in vitro* activity against *M*. *mycetomatis* (EPL-BS0178, EPL-BS0495, EPL-BS0800 and EPLB-BS1025) were screened *in vivo*, it appeared that three of these fenarimol analogues (EPL-BS0178, EPL-BS0495 and EPL-BS1025) showed potent *in vivo* activity. Of these fenarimol analogues, EPL-BS0178 appeared to be the most potent. Interestingly, there appears to be a correlation between the polarity (expressed as logD at pH 7.4, see Fig 10) of the hit compounds and the prolongation of survival in the *G*. *mellonella* larval assay: across the few chemotypes investigated, those compounds with logD values>2.5 were the best performers in this *in vivo* assay model. Correlation between physicochemical properties including polarity, and permeability and tissue distribution *in vivo* are well recognized as a fundamental element of drug discovery [49]. We postulated that the unique nature and structure of the *M*. *mycetomatis* infection, in particular the grain structure, could pose a challenge to drug access which may be regulated by such physiocochemical properties. The inability to create viable grain structures in an *in vitro* setting led us to evaluate this hypothesis *in vivo*. From our data set it appears that polarity and charge may play a role in the ability for a compound to access the fungus in the *in vivo* setting, as shown by the trend (not statistically evaluated) in Fig 10. It is therefore proposed that potency against *M*. *mycetomatis in vivo* may be determined by a play off between the general antifungal activity and the physicochemical properties of the compound, and that this needs to be taken into consideration when seeking future analogues and antifungal agents against *M*. *mycetomatis* [50].

**Fig 11:**
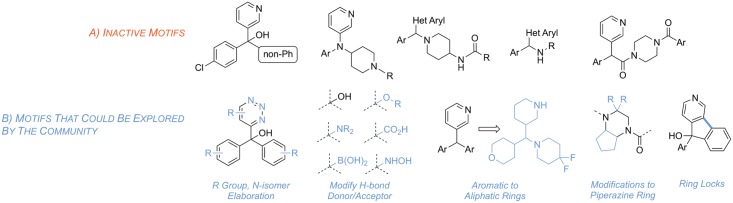
Summary of inactive motifs discovered by screening compounds from the Epichem library (A) and potential motifs for future exploration by the open source community (B).

In light of these findings, it would be interesting to determine if other fenarimol analogues or analogues of 2-aminothiazole increase the therapeutic efficacy against *M*. *mycetomatis* grains *in vitro* and eventually *in vivo*. At present, over 765 of the original fenarimol analogues and a number of 2-aminothiazole analogues series synthesized by other groups have yet to be screened [35, 36]. With these important new results in hand, we propose opening the next stages of this project up to the wider community by adopting an Open Source approach that has previously resulted in effective research consortia in antimalarial drug discovery [51]. We have deposited the data associated with this work in an online database (http://tinyurl.com/MycetomaMols), have started an online discussion area on two websites to gather community expertise (https://github.com/OpenSourceMycetoma and https://www.reddit.com/r/OpenSourceMycetoma/), have opened up the electronic laboratory notebook associated with the chemical resynthesis of fenarimol analogs and have started a social media account for community use and outreach (https://twitter.com/MycetOS). We have used these sites to clarify the current needs of the community, which are i) samples of analogs of the most promising compounds described in this paper and ii) advice on which of the existing compounds to which we have access should next be evaluated *in vitro* and *in vivo*; some directions in the fenarimol library have been suggested (Fig 11). Gathered together, these resources constitute *Open Source Mycetoma* (MycetOS), a project that will adhere to six basic laws of open source research, most importantly that all data and ideas are freely shared, that anyone may participate and that there will be no patents [51]. The authors of this paper, and DND*i* itself, are partners in such an approach, but any other member of the community is free to participate and contribute as an equal partner provided the principle of open work is upheld. We hope that this initiative, coupled with the promising new hits we have reported, will lead to progress in drug discovery for this most neglected of neglected tropical diseases.

## Supporting information

S1 Table Information and data of all compounds tested from the Pathogen Box, Stasis Box and fenarimol analogues. (XLSX)

S1 Text Graphical representation of fenarimol analogues. (DOCX)

S2 Text General method for chemical synthesis of hit compounds and novel analogues. (DOCX)

S3 Text Toxicity of compounds and novel analogues in *G*. *mellonella* larvae. (DOCX)
