# Supplementary material for: Addressing the most neglected diseases through an open research model: The discovery of fenarimols as novel drug candidates for eumycetoma
Source: PLoS Negl Trop Dis. 2018 Apr 26;12(4):e0006437. doi: 10.1371/journal.pntd.0006437 (PMC5940239; doi:10.1371/journal.pntd.0006437)
Supplement: S1 Text — (DOCX) [file pntd.0006437.s002.docx]

Supporting Information 2

for

Addressing the Most Neglected Diseases through an Open Research Model: the Discovery of Fenarimols as Novel Drug Candidates for Eumycetoma

Wilson Lim^1^, Youri Melse^1^, Mickey Konings^1^, Hung Phat Duong^2^, Kimberly Eadie^1^, Benoît Laleu^3^, Ben Perry^4^, Matthew H. Todd^2^, Jean-Robert Ioset^4^, Wendy W.J. van de Sande^1^*

^1^

ErasmusMC

Department of Medical Microbiology and Infectious Diseases

Wytemaweg 80

3015 CE Rotterdam

The Netherlands

^2^School of Chemistry

The University of Sydney

NSW 2006 Sydney

Australia

^3^Medicines for Malaria Venture (MMV),

PO Box 1826,

20, Route de Pré-Bois

1215 Geneva 15,

Switzerland

^4^DNDi,

15 Chemin Louis Dunant,

1202 Geneva,

Switzerland


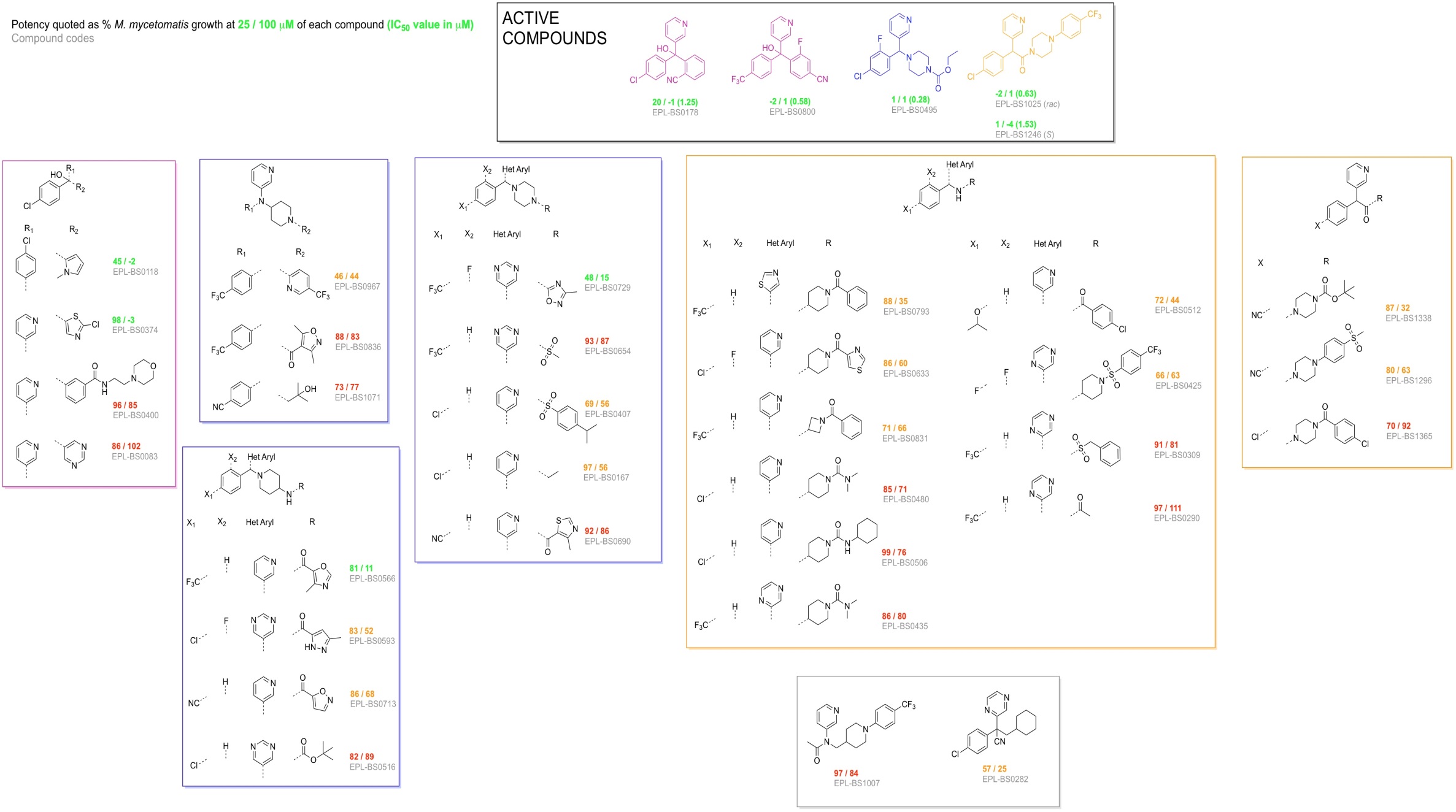
**Graphical representation of fenarimol analogue structures.**
