# Supplementary material for: Addressing the most neglected diseases through an open research model: The discovery of fenarimols as novel drug candidates for eumycetoma
Source: PLoS Negl Trop Dis. 2018 Apr 26;12(4):e0006437. doi: 10.1371/journal.pntd.0006437 (PMC5940239; doi:10.1371/journal.pntd.0006437)
Supplement: S2 Text — (DOCX) [file pntd.0006437.s003.docx]

Supporting Information 3

for

Addressing the Most Neglected Diseases through an Open Research Model: the Discovery of Fenarimols as Novel Drug Candidates for Eumycetoma

Wilson Lim^1^, Youri Melse^1^, Mickey Konings^1^, Hung Phat Duong^2^, Kimberly Eadie^1^, Benoît Laleu^3^, Ben Perry^4^, Matthew H. Todd^2^, Jean-Robert Ioset^4^, Wendy W.J. van de Sande^1^*

^1^

ErasmusMC

Department of Medical Microbiology and Infectious Diseases

Wytemaweg 80

3015 CE Rotterdam

The Netherlands

^2^School of Chemistry

The University of Sydney

NSW 2006 Sydney

Australia

^3^Medicines for Malaria Venture (MMV),

PO Box 1826,

20, Route de Pré-Bois

1215 Geneva 15,

Switzerland

^4^DNDi,

15 Chemin Louis Dunant,

1202 Geneva,

Switzerland

**Table of Contents**

General Methods for Chemical Synthesis S3

**General Methods for Chemical Synthesis**

**Method A, Lithiation**

*n*-Butyllithium (1.5 equiv, 1.6 M solution in hexanes) was added dropwise to a solution of 3-bromopyridine (1.5 equiv) in dried diethyl ether at –78°C. After stirring for 30 min at –78°C, a solution of the 4-bromobenzaldehyde (1 equiv) in dried tetrahydrofuran was added. After stirring for 1 h at –78°C, the reaction was allowed to warm to –40°C, was quenched with a saturated solution of ammonium chloride and extracted with ethyl acetate. The organic phases were combined, dried (MgSO_4_) and concentrated under reduced pressure.

**Method B, Lithiation**

*n*-Butyllithium (2 equiv, 1.6 M solution in hexanes) was added dropwise to a solution of 4-bromobenzotrifluoride (2 equiv) in dried diethyl ether at –78°C. The reaction mixture was stirred for 2 h. A solution of diarylmethanone (1 equiv) in dried tetrahydrofuran was added dropwise at –78°C. The reaction was allowed to warm to rt overnight, was quenched with water and extracted with ethyl acetate. The organic phases were combined, dried (MgSO_4_) and concentrated under reduced pressure.

**Method C, Diarylalcohol Oxidation**

Activated manganese dioxide (3–7 equiv) was added to a solution of diarylalcohol (1 equiv) in dichloromethane. After being heated at reflux for 4 h, the reaction mixture was cooled and filtered through Celite. The filter cake was washed with dichloromethane. The filtrate was concentrated under reduced pressure.

**Method D, Cyanation**

A mixture of bromo-substituted fenarimol (1 equiv), potassium hexacyanoferrate(II) trihydrate (2 equiv), palladium(II) acetate (0.2 equiv), anhydrous sodium carbonate (2 equiv) and anhydrous *N*,*N*-dimethylacetamide was heated under nitrogen at 130°C overnight. After cooling, the reaction mixture was quenched with water. The aqueous layer was extracted with ethyl acetate. The combined organic layers were washed with water, followed by brine, dried (MgSO_4_) and concentrated under reduced pressure.

**(4-Bromo-2-fluorophenyl)(pyridin-3-yl)methanol, S1**

Prepared according to Method A, using 4-bromo-2-fluorobenzaldehyde (5.6 g, 0.027 mol) in tetrahydrofuran (20 mL) to give *the title compound* as a reddish brown waxy solid (7.3 g, 96%), used without further purification. **^1^H NMR** (500 MHz, Acetone-*d*_6_) δ 8.62 (d, *J* = 2.4 Hz, 1H), 8.46 (dd, *J* = 4.8, 1.7 Hz, 1H), 7.74 (ddd, *J* = 7.9, 2.5, 1.3 Hz, 1H), 7.65 (t, *J* = 8.1 Hz, 1H), 7.44 (dd, *J* = 8.4, 1.9 Hz, 1H), 7.37 – 7.24 (m, 2H), 6.13 (s, 1H). **^19^F NMR** (471 MHz, Acetone-*d*_6_) δ –116.62. **LRMS** *m/z* (ESI) 340 (87%), 338 (100%), 284 ([M+H]^+^, 20%), 282 ([M+H]^+^, 18%). **HRMS** (ESI) calcd. for C_12_H_10_^81^BrFNO^+^ 283.99093 and C_12_H_10_^79^BrFNO^+^ 281.99298 ([M+H]^+^), found 283.99035, 281.99242. **IR** (film): *ν*_max_ 3123 (br), 2923, 2853, 1603, 1574, 1479, 1425, 1398 cm^–1^. Spectroscopic data matched those in the literature.[^1^](#_ENREF_1) However, literature characterisation was incomplete.

**(4-Bromo-2-fluorophenyl)(pyridin-3-yl)methanone, S2**

Prepared according to Method C, using activated manganese dioxide (5.8 g, 0.025 mol) and a solution of **S1** (6.7 g, 0.076 mol) in dichloromethane (55 mL). The crude mixture was purified by column chromatography to give *the title compound* as a cream solid (0.83 g, 12%) and recovered **S1** as a reddish brown waxy solid (3.4 g, 50%). **m.p.** 55.7 – 58.3°C, no lit. m.p. **^1^H NMR** (500 MHz, Acetone-*d*_6_) δ 8.98 – 8.93 (m, 1H), 8.84 (dd, *J* = 4.8, 1.7 Hz, 1H), 8.21 – 8.16 (m, 1H), 7.66 – 7.61 (m, 3H), 7.59 (ddd, *J* = 7.9, 4.9, 0.9 Hz, 1H). **^13^C NMR** (126 MHz, Acetone-*d*_6_) δ 190.6, 160.0 (d, *J* = 255.7 Hz), 153.8, 150.4 (d, *J* = 1.7 Hz), 136.5, 132.8, 132.3 (d, *J* = 3.3 Hz), 128.3 (d, *J* = 3.7 Hz), 126.4 (d, *J* = 9.6 Hz), 125.3 (d, *J* = 14.5 Hz), 123.7, 120.0 (d, *J* = 25.2 Hz). **^19^F NMR** (471 MHz, Acetone-*d*_6_) δ –109.99. **LRMS** *m/z* (ESI) 340 (87%), 338 (100%), 304 ([M+Na]^+^, 27%), 302 ([M+Na]^+^, 21%), 282 ([M+H]^+^, 27%), 280 ([M+H]^+^, 23%). **HRMS** (ESI) calcd. for C_12_H_8_^81^BrFNO^+^ 281.97528 and C_12_H_8_^79^BrFNO^+^ 279.97733 ([M+H]^+^), found 281.97476, 279.97677. **IR** (film): *ν*_max_ 3007, 1654 (s), 1584, 1417, 1395 cm^–1^. **Anal.** cald. for C_12_H_7_BrFNO: C 51.46, H 2.52, N 5.00 %, found C 51.35, H 2.11, N 4.89 %. Spectroscopic data matched those in the literature.[^1^](#_ENREF_1) However, literature characterisation was incomplete.

**(4-Bromo-2-fluorophenyl)(pyridin-3-yl)(4-(trifluoromethyl)phenyl)methanol, S3**

Prepared according to Method B, using 4-bromobenzotrifluoride (0.90 mL, 0.0054 mol) in diethyl ether (45 mL), *n*-butyllithium (3.8 mL, 1.6 M solution in hexanes, 0.0053 mol) and a solution of **S2** (0.74 g, 0.0027 mol) in tetrahydrofuran (30 mL). The crude mixture was purified by column chromatography (ethyl acetate/hexane) to give *the title compound* as an orange solid (0.40 g, 35%). **m.p.** 167.1–169.0°C, no lit. m.p. **^1^H NMR** (500 MHz, Acetone-*d*_6_) δ 8.55 (s, 1H), 8.50 (d, *J* = 4.8 Hz, 1H), 7.73 (d, *J* = 8.0 Hz, 3H), 7.62 (d, *J* = 8.2 Hz, 2H), 7.46 (d, *J* = 4.5 Hz, 1H), 7.41 – 7.29 (m, 2H), 6.09 (s, 1H). **^13^C NMR** (126 MHz, Acetone-*d*_6_) δ 160.6 (d, *J* = 252.7 Hz), 150.3, 149.7 (d, *J* = 2.2 Hz), 149.5, 141.4, 135.7 (d, *J* = 1.9 Hz), 133.6 (d, *J* = 11.4 Hz), 131.5 (d, *J* = 3.8 Hz), 130.0 (q, *J* = 32.1 Hz), 129.0 (d, *J* = 1.9 Hz), 128.4 (d, *J* = 3.3 Hz), 125.9 (q, *J* = 3.8 Hz), 125.3 (q, *J* = 271.3 Hz), 123.8, 123.0 (d, *J* = 9.5 Hz), 120.6 (d, *J* = 26.2 Hz), 78.7. **LRMS** *m/z* (ESI) 428 ([M+H]^+^, 91%), 426 ([M+H]^+^, 100%), 348 (22%), 340 (27%), 338 (32%), 333 (17%), 332 (24%). **HRMS** (ESI) calcd. for C_19_H_13_^81^BrF_4_NO^+^ 428.00962 and C_19_H_13_^79^BrF_4_NO^+^ 426.01166 ([M+H]^+^), found 428.00934, 426.01138. **IR** (film): *ν*_max_ 3096 (br), 1601, 1567, 1477, 1419, 1324 cm^–1^. Spectroscopic data matched those in the literature.[^1^](#_ENREF_1) However, literature characterisation was incomplete.

**3-Fluoro-4-(hydroxy(pyridin-3-yl)(4-(trifluoromethyl)phenyl)methyl)benzonitrile, S4, EPL-BS0800**

Prepared according to Method D, using **S3** (0.20 g, 0.45 mmol), potassium hexacyanoferrate(II) trihydrate (0.42 g, 0.95 mmol), palladium acetate (0.031 g, 0.062 mmol), anhydrous sodium carbonate (0.057 g, 0.93 mmol) and anhydrous *N*,*N*-dimethylacetamide (2.3 mL). The crude product was purified with column chromatography (hexane/ethyl acetate) to obtain *the title compound* as a pale straw-coloured fine solid (55 mg, 32%). **m.p.** 129.5–132.1°C, no lit. m.p.  **^1^H NMR** (500 MHz, Acetone-*d*_6_) δ 8.57 (d, *J* = 2.5 Hz, 1H), 8.53 (dd, *J* = 4.8, 1.6 Hz, 1H), 7.82 (t, *J* = 8.0 Hz, 1H), 7.77 – 7.69 (m, 4H), 7.66 – 7.58 (m, 3H), 7.38 (ddd, *J* = 8.1, 4.7, 0.8 Hz, 1H), 6.22 (s, 1H). **^13^C NMR** (126 MHz, Acetone-*d*_6_) δ 160.2 (d, *J* = 251.0 Hz), 149.8, 149.7 (d, *J* = 2.3 Hz), 140.8, 139.5 (d, *J* = 11.4 Hz), 135.7 (d, *J* = 2.1 Hz), 131.1 (d, *J* = 3.8 Hz), 130.4 (q, *J* = 32.2 Hz), 129.5 (d, *J* = 3.7 Hz), 129.0 (d, *J* = 1.9 Hz), 126.0 (q, *J* = 3.8 Hz), 125.2 (q, *J* = 271.2 Hz), 123.9, 121.0 (d, *J* = 26.8 Hz), 117.9 (d, *J* = 2.7 Hz), 114.6 (d, *J* = 10.0 Hz), 78.9 (1 obscured signal). **^19^F NMR** (471 MHz, Acetone-*d*_6_) δ –63.07, –104.20. **HRMS** (ESI) calcd. for C_20_H_13_F_4_N_2_O^+^ 373.09640 ([M+H]^+^), found 373.09610. **IR** (film): *ν*_max_ 3077 (br), 2241, 1617, 1562, 1490, 1411, 1325 cm^–1^. Spectroscopic data matched those in the literature.[^1^](#_ENREF_1) However, literature characterisation was incomplete.

**(4-Bromophenyl)(pyridin-3-yl)methanol, S5**

Prepared according to Method A, using 4-bromobenzaldehyde (10 g, 0.054 mol) to give *the title compound* as an orange solid (13 g, 95%), used without further purification. **^1^H NMR** (500 MHz, Acetone-*d*_6_) δ 8.63 (d, *J* = 2.3 Hz, 1H), 8.44 (dd, *J* = 4.7, 1.7 Hz, 1H), 7.77 – 7.69 (m, 1H), 7.55 – 7.47 (m, 2H), 7.44 – 7.36 (m, 2H), 7.30 (ddd, *J* = 7.8, 4.8, 0.9 Hz, 1H), 5.92 (s, 1H). **^13^C NMR** (126 MHz, Acetone-*d*_6_) δ 148.5, 148.3, 144.2, 140.2, 133.7, 131.3, 128.4, 123.2, 120.5, 72.5. **LRMS** *m/z* (ESI) 322 (100%), 320 (95%), 266 ([M+H]^+^, 28%), 264 ([M+H]^+^, 27%). **HRMS** (ESI) calcd. for C_12_H_10_^81^BrNONa^+^ 287.98230, C_12_H_10_^79^BrNONa^+^ 285.98435 ([M+Na]^+^), found 287.98208, 285.98414. **IR** (film): *ν*_max_ 3149 (br), 2856, 1588, 1578, 1486, 1474, 1424, 1396 cm^–1^. No spectroscopic data available for comparison.

**(4-Bromophenyl)(pyridin-3-yl)methanone, S6**

Prepared according to Method C, using activated manganese dioxide (21 g, 0.24 mol) and a solution of **S5** (12 g, 0.045 mol) in dichloromethane (75 mL). The crude mixture was purified by column chromatography to give *the title compound* as a cream solid (7.8 g, 69 %) and recovered **S5** as an orange solid (1.4 g, 12%). **m.p.** 123.3–125.3°C, no lit. m.p. **^1^H NMR** (500 MHz, Acetone-*d*_6_) δ 8.94 (dd, *J* = 2.3, 0.9 Hz, 1H), 8.83 (dd, *J* = 4.9, 1.7 Hz, 1H), 8.15 (dt, *J* = 7.9, 2.0 Hz, 1H), 7.86 – 7.69 (m, 4H), 7.58 (ddd, *J* = 7.9, 4.8, 0.9 Hz, 1H). **^13^C NMR** (126 MHz, Acetone-*d*_6_) δ 193.5, 153.0, 150.4, 136.8, 136.0, 132.8, 131.9, 131.6, 127.4, 123.4. **LRMS** *m/z* (ESI) 286 ([M+Na]^+^, 100%), 284 ([M+Na]^+^, 79%), 264 ([M+H]^+^, 45%), 262 ([M+H]^+^, 44%). **HRMS** (ESI) calcd. for C_12_H_8_^81^BrNONa^+^ 285.96665 and C_12_H_8_^79^BrNONa^+^ 283.96870 ([M+Na]^+^), found 285.96649, 283.96851. **IR** (film): *ν*_max_ 1650 (s), 1581, 1479, 1414, 1393, 1337 cm^–1^. **Anal.** cald. for C_12_H_8_BrNO: C 54.99, H 3.08, N 5.34 %, found C 54.98, H 2.84, N 5.26 %. No spectroscopic data available for comparison.

**(4-Bromophenyl)(pyridin-3-yl)(4-(trifluoromethyl)phenyl)methanol, S7**

Prepared according to Method B, using 4-bromobenzotrifluoride (1.5 mL, 0.011 mol) in diethyl ether (145 mL), *N*-butyllithium (6.6 mL, 1.6 M solution in hexanes, 0.011 mol) and a solution of **S6** (1.5 g, 0.0053 mol) in tetrahydrofuran (90 mL). The crude mix was purified by column chromatography (ethyl acetate/hexane) to give *the title compound* as a straw coloured solid (0.14 g, 5.8%) and recovered **S6** as a cream solid (0.18 g, 12%). **^1^H NMR** (500 MHz, Acetone-*d*_6_) δ 8.52 (dd, *J* = 2.5, 0.8 Hz, 1H), 8.50 (dd, *J* = 4.7, 1.6 Hz, 1H), 7.81 – 7.75 (m, 1H), 7.69 (tdd, *J* = 8.7, 2.6, 1.6 Hz, 2H), 7.63 – 7.48 (m, 4H), 7.36 (ddd, *J* = 8.0, 4.8, 0.9 Hz, 1H), 7.32 – 7.19 (m, 2H). **^13^C NMR** (126 MHz, Acetone-*d*_6_) δ 150.1, 149.5, 148.7 (d, *J* = 3.5 Hz), 146.5 (d, *J* = 3.9 Hz), 142.7 (d, *J* = 3.6 Hz), 136.0, 132.7, 132.0, 130.8, 130.7 (q, *J* = 31.9 Hz), 129.9, 125.3 (q, *J* = 271.6 Hz), 125.1 (q, *J* = 3.9 Hz), 124.9 (q, *J* = 4.0 Hz), 123.8, 122.1, 80.4 (d, *J* = 10.3 Hz). **^19^F NMR** (471 MHz, Acetone-*d*_6_) δ -63.04. **LRMS** *m/z* (ESI) 410 ([M+H]^+^, 94%), 408 ([M+H]^+^, 100%). **HRMS** (ESI) calcd. for C_19_H_14_^81^BrF_3_NO^+^ 410.01904 and C_19_H_14_^79^BrF_3_NO^+^ 408.02109 ([M+H]^+^), found 410.01868, 408.02072. **IR** (film): *ν*_max_ 3066 (br), 2782, 1589, 1486, 1420, 1394, 1326 cm^–1^. **Anal.** cald. C 67.80, H 3.70, N 7.84 %, found C 67.50, H 3.03, N 7.82 %. All data support isolation of a pure product, but the %H value is slightly outside tolerance limits that is not easily accounted for by typical solvent or water inclusion. No spectroscopic data available for comparison.

**4-(Hydroxy(pyridin-3-yl)(4-(trifluoromethyl)phenyl)methyl)benzonitrile, S8**

Prepared according to the cyanation general reaction, using **S7** (150 mg, 0.42 mmol), potassium hexacyanoferrate(II) trihydrate (0.35 g, 0.79 mmol), palladium acetate (0.031 g, 0.062 mmol), anhydrous sodium carbonate (63 mg, 1.0 mmol) and anhydrous *N*,*N*-dimethylacetamide (1.8 mL). The crude product was purified with column chromatography (hexane/ethyl acetate) to obtain *the title compound* as a pale straw coloured fine solid (69 mg, 53%). **^1^H NMR** (500 MHz, Acetone-*d*_6_) δ 8.55 – 8.47 (m, 2H), 7.89 – 7.75 (m, 3H), 7.70 (ddd, *J* = 8.2, 4.2, 2.1 Hz, 2H), 7.66 – 7.47 (m, 4H), 7.37 (dd, *J* = 8.0, 4.8 Hz, 1H). **^13^C NMR** (126 MHz, Acetone-*d*_6_) δ 152.1 (d, *J* = 5.0 Hz), 150.1, 149.7, 148.1 (d, *J* = 5.1 Hz), 142.3 (d, *J* = 5.2 Hz), 136.1, 132.9, 132.7, 130.9 (q, *J* = 32.0 Hz), 130.0, 129.6, 125.3 (q, *J* = 3.8 Hz), 125.2 (q, *J* = 271.7 Hz), 124.9 (q, *J* = 3.9 Hz), 123.9, 119.1, 112.3, 80.5 (d, *J* = 10.8 Hz). **LRMS** *m/z* (ESI) 707 ([2M–H]^–^, 88%), 353 ([M–H]^–^, 100%). **HRMS** (ESI) calcd. for C_20_H_14_F_3_N_2_O^+^ 355.10582, found 355.10543 ([M+H]^+^). **IR** (film): *ν*_max_ 3055 (br), 2230 (w), 1606, 1592, 1578, 1501, 1476, 1420, 1327 cm^–1^. No spectroscopic data available for comparison.

**(4-Chloro-2-fluorophenyl)(pyridin-3-yl)methanol, S9**

Prepared according to Method A, using 4-chloro-2-fluorobenzaldehyde (4.4 g, 0.027 mol) to give *the title compound* as a reddish orange waxy solid (5.6 g, 88%), used without further purification. **^1^H NMR** (500 MHz, Acetone-*d*_6_) δ 8.62 (d, *J* = 2.4 Hz, 1H), 8.46 (dd, *J* = 4.7, 1.7 Hz, 1H), 7.92 – 7.57 (m, 2H), 7.51 – 7.26 (m, 2H), 7.20 (m, 1H), 6.14 (s, 1H). **^13^C NMR** (126 MHz, Acetone-*d*_6_) δ 160.3 (d, *J* = 248.7 Hz), 149.6, 149.2 (d, *J* = 1.6 Hz), 139.9, 134.6, 134.3 (d, *J* = 10.5 Hz), 131.7 (d, *J* = 13.7 Hz), 129.9 (d, *J* = 5.2 Hz), 125.7 (d, *J* = 3.6 Hz), 124.2, 116.6 (d, *J* = 25.4 Hz), 67.6. **^19^F NMR** (471 MHz, Acetone-*d*_6_) δ –116.82. **LRMS** *m/z* (ESI) 296 (27%), 294 (100%), 240 ([M+H]^+^, 2%), 238 ([M+H]^+^, 8%). **HRMS** (ESI) calcd. for C_12_H_10_^37^ClFNO^+^ 240.04054 and C_12_H_10_^35^ClFNO^+^ 238.04350 ([M+H]^+^), found 240.04000, 238.04295. **IR** (film): *ν*_max_ 3113 (br), 2929, 1609, 1578, 1482, 1426, 1402 cm^–1^. No spectroscopic data available for comparison.

***tert*-Butyl 4-((4-chloro-2-fluorophenyl)(pyridin-3-yl)methyl)piperazine-1-carboxylate, S10**

Thionyl chloride (3.6 mL, 0.047 mol) was added to a solution of **S9** (5.0 g, 0.023 mol) in dichloromethane (100 mL) at 0°C. The reaction mixture was allowed to warm to room temperature over 2 h, quenched with a saturated solution of sodium carbonate (100 mL) and extracted with dichloromethane (3 × 100 mL). The organic phases were combined, washed with brine (2 × 75 mL), dried (MgSO_4_) and concentrated under reduced pressure to give the aryl chloride intermediate **S9a** as a crude light brown oil (3.3 g, 66%), used without further purification.

Anhydrous triethylamine (3.6 mL, 0.026 mol) and dried potassium iodide (1 small spatula tip) were added to a solution of **S9a** (3.0 g) and *tert*-butyl piperazine-1-carboxylate (3.6 g, 0.019 mol) in anhydrous acetonitrile (25 mL). After being heated at 80°C for 48 h, the reaction mixture was cooled and concentrated under reduced pressure. The concentrated residue was partitioned between dichloromethane (100 mL) and a saturated solution of sodium carbonate (100 mL). The aqueous layer was extracted with dichloromethane (2 × 50 mL). The organic phases were combined, dried (MgSO_4_) and concentrated under reduced pressure to give a crude orange oil. The crude oil was purified with column chromatography to give *the title compound* as an amber resin (3.5 g, 75%). **^1^H NMR** (200 MHz, Chloroform-*d*) δ 8.59 (d, *J* = 2.2 Hz, 1H), 8.41 (dd, *J* = 4.8, 1.6 Hz, 1H), 7.64 (dt, *J* = 7.9, 2.0 Hz, 1H), 7.46 (t, *J* = 8.0 Hz, 1H), 7.16 (ddd, *J* = 7.9, 4.8, 0.9 Hz, 1H), 7.12 – 6.99 (m, 1H), 6.96 (dd, *J* = 9.9, 2.1 Hz, 1H), 4.64 (s, 1H), 3.38 (t, *J* = 5.1 Hz, 4H), 2.69 – 2.18 (m, 4H), 1.38 (s, 9H). **^13^C NMR** (75 MHz, Chloroform-*d*) δ 160.3 (d, *J* = 250.1 Hz), 154.6, 149.6, 148.9, 136.3, 135.4, 133.8 (d, *J* = 10.5 Hz), 129.5 (d, *J* = 4.7 Hz), 126.8 (d, *J* = 12.7 Hz), 125.1 (d, *J* = 3.4 Hz), 123.6, 116.5 (d, *J* = 26.1 Hz), 79.6, 64.3, 51.4, 28.4 (1 obscured signal). **LRMS** 430 ([M+Na]^+^, 35%), 428 ([M+Na]^+^, 100%), 408 ([M+H]^+^, 7%), 406 ([M+H]^+^, 19%). **HRMS** (ESI) calcd. for C_21_H_25_^37^ClFN_3_O_2_Na^+^ 430.14875 and C_21_H_25_^35^ClFN_3_O_2_Na^+^ 428.15170 ([M+Na]^+^), found 430.14882, 428.15181. **IR** (film): *ν*_max_ 2974, 2814, 1688 (s), 1608, 1578, 1480, 1420, 1365 cm^–1^. No spectroscopic data available for comparison.

**Ethyl 4-((4-chloro-2-fluorophenyl)(pyridin-3-yl)methyl)piperazine-1-carboxylate, S11, EPL-BS0495**

Trifluoroacetic acid (6 mL, 0.080 mol) was added to a solution of **S10** (3.2 g, 0.0078 mol) and methanol (11 drops) in dichloromethane (35 mL) at 0°C. After stirring at 0°C for 1 h, the reaction mixture was allowed to warm to room temperature overnight, concentrated under reduced pressure to give the crude trifluoroacetic acid salt intermediate **S10a** (6.4 g), used without further purification.

Anhydrous triethylamine (25 mL, 0.18 mol) and ethyl chloroformate (0.75 mL 0.0075 mol) were added to a solution of **S10a** (6.4 g) in dichloromethane (50 mL) at 0°C. The reaction mixture was allowed to warm to rt overnight, quenched with a saturated solution of ammonium chloride (30 mL) and extracted with dichloromethane (3 × 30 mL). The organic phases were combined, dried (MgSO_4_) and concentrated under reduced pressure. The crude product was purified with column chromatography (ethyl acetate/hexane) to give *the title compound* as an amber resin (2.0 g, 65%). **^1^H NMR** (500 MHz, Chloroform-*d*) δ 8.63 (d, *J* = 2.2 Hz, 1H), 8.47 (dd, *J* = 4.8, 1.6 Hz, 1H), 7.68 (dt, *J* = 7.9, 2.0 Hz, 1H), 7.50 (t, *J* = 8.0 Hz, 1H), 7.22 (ddd, *J* = 7.9, 4.7, 0.8 Hz, 1H), 7.13 (dd, *J* = 8.4, 2.0 Hz, 1H), 7.03 (dd, *J* = 9.9, 2.1 Hz, 1H), 4.69 (s, 1H), 4.10 (q, *J* = 7.1 Hz, 2H), 3.47 (t, *J* = 5.1 Hz, 4H), 2.55 – 2.07 (m, 4H), 1.22 (t, *J* = 7.1 Hz, 3H). **^13^C NMR** (126 MHz, Chloroform-*d*) δ 160.4 (d, *J* = 250.0 Hz), 155.5, 149.8, 149.1, 136.4, 135.5, 134.0 (d, *J* = 10.6 Hz), 129.6 (d, *J* = 4.7 Hz), 126.8 (d, *J* = 12.8 Hz), 125.2 (d, *J* = 3.5 Hz), 123.8, 116.7 (d, *J* = 26.0 Hz), 64.4 (d, *J* = 1.7 Hz), 61.5, 51.5, 43.8, 14.8. **LRMS** *m/z* (ESI) 402 ([M+Na]^+^, 37%), 400 ([M+Na]^+^, 100%). **HRMS** (ESI) calcd. for C_19_H_21_^37^ClFN_3_O_2_Na^+^ 402.11745 and C_19_H_21_^35^ClFN_3_O_2_Na^+^ 400.12040 ([M+Na]^+^), found 402.11709 and 400.12007. **IR** (film): *ν*_max_ 2923, 2853, 1603, 1574, 1479, 1425, 1398 cm^–1^. No spectroscopic data available for comparison.

**Cetirizine hydrochloride, S12**

Purchased from Sigma Aldrich. **^1^H NMR** (500 MHz, Deuterium Oxide) δ 7.63 – 7.59 (m, 2H), 7.58 – 7.44 (m, 5H), 7.45 – 7.37 (m, 2H), 5.38 (s, 1H), 4.24 (s, 2H), 4.07 – 3.83 (m, 2H), 3.72 (s, 4H), 3.58 – 3.52 (m, 2H), 3.49 (s, 4H). **^13^C NMR** (126 MHz, Deuterium Oxide) δ 174.5, 135.1, 133.7, 132.6, 129.9, 129.8, 129.5, 127.9, 74.7, 67.5, 63.9, 55.9, 49.1, 48.3 (1 obscured signal). **LRMS** *m/z* (ESI) 413 ([M+Na]^+^, 38%), 411 ([M+Na]^+^, 100%), 391 ([M+H]^+^, 28%), 389 ([M+H]^+^, 76%), 203 ([M–(C_8_H_15_N_2_O_3_)^–^]^+^, 15%), 201 ([M–(C_8_H_15_N_2_O_3_)^–^]^+^, 40%). **HRMS** (ESI) calcd. for C_21_H_26_^37^ClN_2_O_3_^+^ 391.16025 and C_21_H_26_^35^ClN_2_O_3_^+^ 389.16320 ([M+H]^+^), found 391.15987, 389.16286. Spectroscopic data matched those in the literature.[^2-3^](#_ENREF_2)

**References**

1. Keenan, M.; Abbott, M. J.; Alexander, P. W.; Armstrong, T.; Best, W. M.; Berven, B.; Botero, A.; Chaplin, J. H.; Charman, S. A.; Chatelain, E.; von Geldern, T. W.; Kerfoot, M.; Khong, A.; Nguyen, T.; McManus, J. D.; Morizzi, J.; Ryan, E.; Scandale, I.; Thompson, R. A.; Wang, S. Z.; White, K. L., Analogues of Fenarimol Are Potent Inhibitors of Trypanosoma cruzi and Are Efficacious in a Murine Model of Chagas Disease. *J. Med. Chem.* **2012,** *55* (9), 4189–4204.

2. Dyakonov, T.; Muir, A.; Nasri, H.; Toops, D.; Fatmi, A., Isolation and Characterization of Cetirizine Degradation Product: Mechanism of Cetirizine Oxidation. *Pharmaceutical Research* **2010,** *27* (7), 1318-1324.

3. Tan, Z.-R.; Ouyang, D.-S.; Zhou, H.-H.; Zhou, G.; Wang, L.-S.; Wang, D.; Li, Z., Sensitive bioassay for the simultaneous determination of pseudoephedrine and cetirizine in human plasma by liquid-chromatography–ion trap spectrometry. *J. Pharm. Biomed. Anal.* **2006,** *42* (2), 207-212.
