# Supplementary material for: Addressing the most neglected diseases through an open research model: The discovery of fenarimols as novel drug candidates for eumycetoma
Source: PLoS Negl Trop Dis. 2018 Apr 26;12(4):e0006437. doi: 10.1371/journal.pntd.0006437 (PMC5940239; doi:10.1371/journal.pntd.0006437)
Supplement: S3 Text — (DOCX) [file pntd.0006437.s004.docx]

Supporting Information 4

for

Addressing the Most Neglected Diseases through an Open Research Model: the Discovery of Fenarimols as Novel Drug Candidates for Eumycetoma

Wilson Lim^1^, Youri Melse^1^, Mickey Konings^1^, Hung Phat Duong^2^, Kimberly Eadie^1^, Benoît Laleu^3^, Ben Perry^4^, Matthew H. Todd^2^, Jean-Robert Ioset^4^, Wendy W.J. van de Sande^1^*

^1^

ErasmusMC

Department of Medical Microbiology and Infectious Diseases

Wytemaweg 80

3015 CE Rotterdam

The Netherlands

^2^School of Chemistry

The University of Sydney

NSW 2006 Sydney

Australia

^3^Medicines for Malaria Venture (MMV),

PO Box 1826,

20, Route de Pré-Bois

1215 Geneva 15,

Switzerland

^4^DNDi,

15 Chemin Louis Dunant,

1202 Geneva,

Switzerland

**Toxicity of the compounds tested**

As shown in the figures and table, none of the compounds tested appeared to be toxic at a concentration of 20 µM/larvae. In the table we depicted the overall survival at day 10. A compound which had an overall survival of >80% was considered non-toxic. To determine if the survival curves were comparable when larvae were injected with water only, a Log-Rank test was performed. As can be seen in the table, none of the survival lines differed significantly.





| Compound | Name | Derivative | Survival | P-value |
| --- | --- | --- | --- | --- |
| MMV688774 | Posaconazole |  | 90.000% | 0.8265 |
| MMV688942 | Bitertanol |  | 86.667% | 0.4605 |
| MMV688943 | Difenoconazol |  | 85.714% | 0.5268 |
| MMV021057 | Azoxystrobin |  | 80.000% | 0.2030 |
| MMV688754 | Trifloxystrobin |  | 83.333% | 0.4895 |
| MMV689244 | Fenarimol | EPL-BS1246 | 90.909% | 0.9537 |
|  |  | EPL-BS0178 | 80.000% | 0.1050 |
|  |  | EPL-BS0495 | 100.000% | 0.3760 |
|  |  | EPL-BS0800 | 100.000% | 0.2696 |
|  |  | EBP-BS1025 | 80.000% | 0.2067 |
| MMV675968 |  |  | 87.500% | 0.4720 |
| MMV687807 |  |  | 89.655% | 0.6748 |
| MMV022478 |  |  | 92.655% | 0.9076 |
| MMV006357 |  |  | 87.500% | 0.6197 |
